# Artificial intelligence for pneumonia diagnosis: a systematic review of multimodal learning, fusion strategies, and explainability

**DOI:** 10.3389/fdgth.2026.1949796

**Published:** 2026-09-28

**Authors:** K. G. Sruthy, T. M. Navamani

**Affiliations:** School of Computer Science and Engineering (SCOPE), Vellore Institute of Technology (VIT), Vellore, Tamil Nadu, India

**Keywords:** artificial intelligence, explainable artificial intelligence, medical image analysis, multimodal deep learning, multimodal fusion, pneumonia diagnosis

## Abstract

Pneumonia remains a major global public health challenge, particularly among children, older adults, and individuals with weakened immune systems. Although deep learning has made great progress in automated pneumonia diagnosis, most current methods are unimodal, using only chest X-ray images. This results in limited robustness, cross-institutional generalization and clinical applicability. Moreover, the current reviews mainly focus on summarising single data modalities or model architectures and lack comparative analysis of multimodal fusion strategies, explainability methods, benchmark datasets, and new artificial intelligence paradigms. To fill these gaps, this systematic review offers a common analytical framework for systematically comparing multimodal deep learning architectures, fusion strategies, explainability methods, benchmark datasets, and emerging technologies in the field of artificial intelligence for pneumonia diagnosis. Research articles were systematically reviewed to identify the latest developments, technological challenges, and future research directions. Evidence suggests that multimodal models can provide information from different sources and may improve diagnostic performance in specific settings. However, direct comparisons between studies are limited by differences in datasets, clinical tasks, and validation protocols. Attention-based and hybrid fusion strategies can learn useful information from various modalities. Explainable artificial intelligence methods provide mechanisms for improving the transparency and interpretability of model predictions.The clinical translation is still limited, however, due to the lack of large-scale multimodal datasets, data heterogeneity, missing modalities, inconsistent evaluation protocols, and lack of external validation. In summary, this review summarizes the current research trends, outlines important technological challenges, and provides a research agenda for the future to build strong, interpretable, and clinically viable artificial intelligence systems for next-generation pneumonia diagnosis.

## 1 Introduction

Pneumonia continues to be a significant public health problem worldwide, especially in young children, the elderly, and immunocompromised patients. The World Health Organization still considers it to be one of the most important causes of infection-related death in the world (1). Although supportive care and treatment have improved, timely and accurate diagnosis is still important to minimise complications and maximise patient management. Diagnosis of pneumonia, nonetheless, is difficult as it involves clinical history, medical imaging, laboratory investigations and patient-specific risk factors. In addition, clinical and radiological features of pneumonia are not specific to different etiologies, and radiological features, such as consolidation, are not specific to pneumonia (2). The main etiological groups of pneumonia are depicted in Figure 1, and the pathogens, imaging characteristics, and clinical features are matched. The high similarity and complexity of imaging findings and clinical features across different pneumonia etiologies address the challenges of pneumonia diagnosis and the need to develop advanced artificial intelligence models that can combine complementary multimodal information.

**Figure 1:**
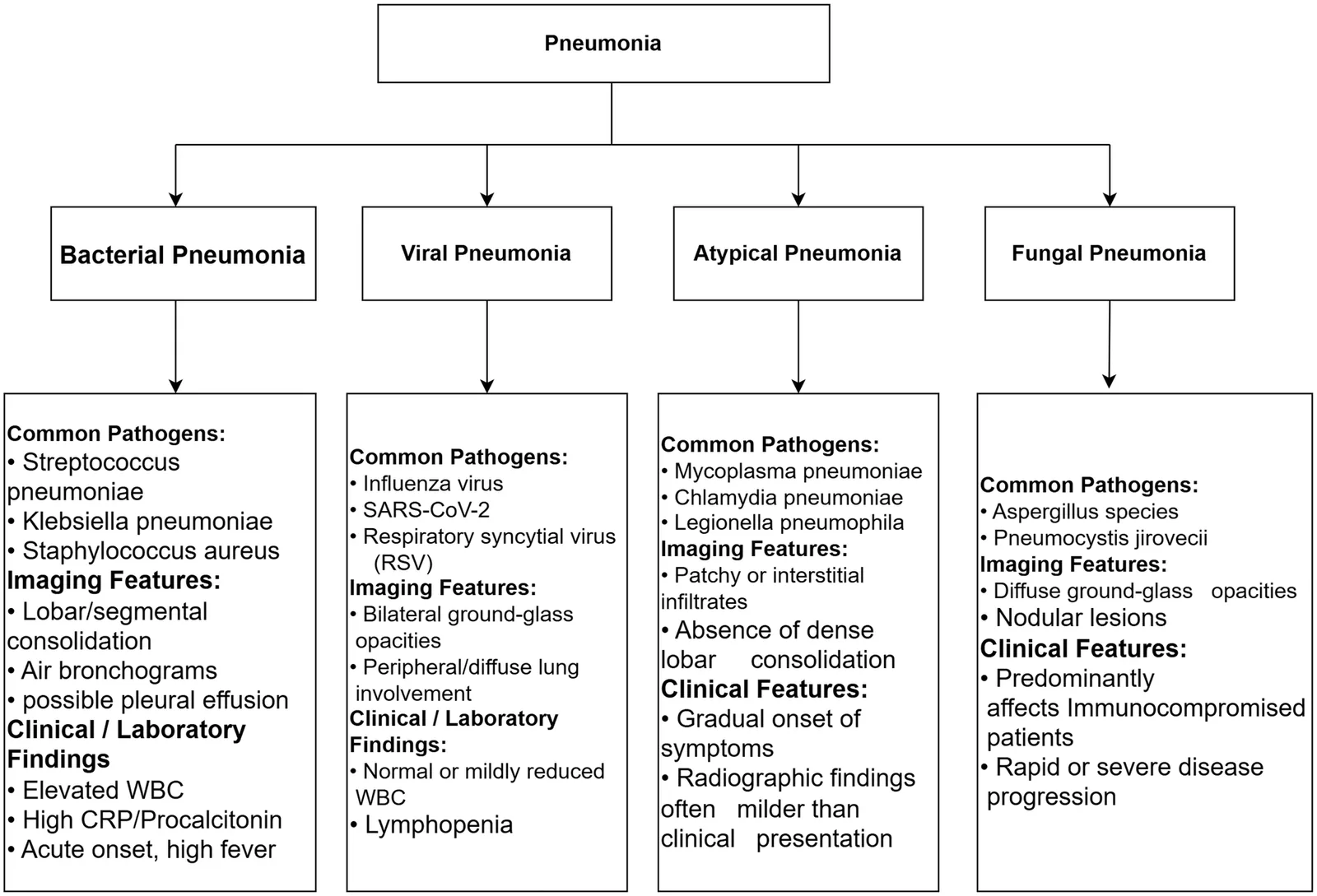
Overview of the major types of pneumonia, illustrating their associated pathogens, characteristic imaging findings, and key clinical features.

Pneumonia is automatically diagnosed using Artificial Intelligence (AI) and Deep Learning (DL). These methods have improved clinical decision support, lesion segmentation, and disease classification. Convolutional Neural Network (CNN)-based models, such as ResNet, DenseNet, and U-Net variants, have reported high performance in lesion segmentation and pneumonia classification from CT and chest X-ray images (3–5). Recently, transformer-based and hybrid CNN-Transformer models have been introduced to improve diagnostic performance further. These models effectively capture both local and global image representations (6, 7). However, current deep learning models are mostly limited to a single data modality, mainly chest X-ray images. These unimodal models have been highly accurate in experimental settings but have not been widely applied in clinical settings due to domain shift, dataset bias, limited external validation, and a lack of ability to incorporate complementary clinical information from multiple healthcare data sources (8).

Several influential studies show how AI-based pneumonia detection has developed over time. Wang et al. (9) introduced the large-scale ChestX-ray8 benchmark and a weakly supervised CNN model for thoracic disease classification and localization. In the same year, Rajpurkar et al. (10) developed CheXNet, a DenseNet-121-based model that achieved radiologist-level performance for pneumonia detection and used class activation mapping for visual localization. Kundu et al. (11) later used a weighted ensemble of GoogLeNet, ResNet-18, and DenseNet-121 for pneumonia classification, showing the potential of model-level fusion. More recently, Shakeri and Far (12) compared pretrained DenseNet and ResNet architectures for the same task. Together, these studies show the shift from large-scale CNN-based classification to transfer learning and ensemble methods. They also provide a foundation for later developments in pneumonia diagnosis.

The shortcomings of unimodal models have led to an increasing interest in multimodal deep learning and advanced learning paradigms that combine complementary information from multiple healthcare data sources in recent years. Some methods, such as sparse deep learning and semi-supervised domain adaptation, have enhanced the model’s robustness and generalization across institutions by leveraging chest CT and chest X-ray data (13, 14). More recently, multimodal approaches that integrate medical imaging with plasma proteomics, laboratory markers, clinical data, and radiology reports have improved disease characterisation, prognosis, and diagnostic accuracy (15–17). In addition to radiological imaging, respiratory sound analysis and lung ultrasonography have become useful supporting data sources for the diagnosis of pneumonia.

Explainable AI techniques also make model predictions easier to interpret, allowing clinicians to better understand how diagnostic decisions are reached. This can strengthen confidence in the use of AI in clinical context. However, before we can use multimodal deep learning for pneumonia detection, there are still a lot of challenges to be solved. These problems include the scarcity of multimodal datasets with appropriate annotations, the variability and incompleteness of data modality, the inability to explain the model, and the lack of external validation (18, 19). Furthermore, there is currently no unified framework that makes it easier to compare multimodal architectures, fusion strategies, benchmark datasets, and explainability techniques in a systematic manner. As a result, obtaining an overall understanding of recent advances in this field remains challenging. Existing reviews have either focused on certain deep learning architectures or on a single data modality. A comprehensive systematic review that provides an integrated analytical comparison of multimodal deep learning strategies, challenges, and future research directions is currently lacking.

Although multimodal learning, fusion strategies, and explainability are the main focus of this review, unimodal studies are also included. They help describe the strengths and limitations of individual healthcare data modalities and provide context for the shift from single-modality models to systems that combine different types of healthcare data. Emerging approaches, including foundation models, vision-language models, self-supervised learning, federated learning, and generative AI, are also covered. These methods may support multimodal pneumonia diagnosis and help address issues such as limited annotated data, data heterogeneity, privacy, missing modalities, and generalization across healthcare settings. The review also examines current challenges and areas that require further research.The objective of this review is to systematically analyze the available data on the use of AI-driven techniques for pneumonia diagnosis. It focuses on multimodal learning, deep learning architectures, fusion strategies, explainability, diagnostic performance, and clinical applicability. The integration of different health care data modalities, AI structures, fusion techniques, and methods for interpreting the outcomes will all be studied. To achieve this objective, the review addresses the following research questions:

RQ1: What healthcare data modalities and AI architectures have been used for pneumonia diagnosis and related diagnostic tasks?
RQ2: What multimodal fusion strategies have been used to combine imaging, clinical, laboratory, respiratory, and molecular data?
RQ3: What explainability techniques have been used to interpret AI models for the diagnosis of pneumonia, and how have they been evaluated?
RQ4: What diagnostic performance has been reported by the reviewed AI approaches, and what factors should be considered when comparing their performance under different conditions?
RQ5: What are the main challenges limiting the clinical applicability, generalizability, and reliability of AI-based pneumonia diagnosis, and how are these barriers being addressed by current research trends?

This review differs from previous reviews in that it compares new AI technologies, deep learning models, explainability techniques, benchmarks, and fusion methodologies. It also examines methodological and clinical challenges that may affect the generalizability and use of AI-based approaches for pneumonia diagnosis in clinical practice. The key findings of this review are listed below:

- Critically summarizes recent deep learning methods for pneumonia diagnosis, focusing on multimodal learning that combines various healthcare data modalities such as medical imaging, clinical and laboratory data, respiratory sounds, lung ultrasonography, and multi-omics data.
- Compares multimodal fusion strategies such as early fusion, hybrid fusion, late fusion, and attention-based fusion techniques and discusses their advantages, disadvantages, and potential clinical applications.
- Critically reviews and compares the current deep learning architectures, benchmark datasets, evaluation metrics, and explainable artificial intelligence techniques, and offers an integrated view of the current technological advances towards trustworthy AI-assisted pneumonia diagnosis.
- Recognizes the key challenges of multimodal deep learning, such as data heterogeneity, missing modalities, multimodal alignment, annotation quality, explainability, and limited external clinical validation, which hinder the deployment of multimodal deep learning in real-world settings.
- Identifies future research directions and presents a technology roadmap by examining emerging artificial intelligence approaches and multimodal learning strategies for developing robust, interpretable, and clinically applicable pneumonia diagnostic systems.

## 2 Methods

This section outlines the methodology used to identify, screen, and synthesize studies on artificial intelligence for pneumonia diagnosis. The following subsections present the search strategy, inclusion criteria, study selection process, and data extraction methods. This review adhered to the PRISMA 2020 guidelines (20).

### 2.1 Literature search strategy

Scopus, PubMed, IEEE Xplore, and Web of Science are the four scientific databases that were searched. These databases were selected because they contain articles about engineering and medicine that are relevant to pneumonia detection. Articles published between January 2023 and July 2026 were included. Moreover, the search terms were grouped into four categories: (i) disease-related terms, (ii) AI techniques, (iii) healthcare data modalities, and (iv) multimodal integration strategies. Boolean operators (AND/OR) were used to construct database-specific search queries. The search was limited to English-language, peer-reviewed journal articles. The database-specific search strategies are presented in Table 1.

**Table 1:** Database-specific search strategies used for the systematic literature search.

Database	Search query
Scopus	TITLE-ABS-KEY (pneumonia) AND TITLE-ABS-KEY (“deep learning” OR “machine learning” OR “artificial intelligence” OR CNN OR transformer OR “neural network*”) AND TITLE-ABS-KEY (“chest x-ray” OR radiograph OR CT OR imaging OR clinical OR laboratory OR EHR OR biomarker OR “vital signs” OR audio OR “lung sound” OR auscultation OR multimodal OR “data fusion”) AND (PUBYEAR > 2022) AND (PUBYEAR < 2027) AND (LIMIT-TO (LANGUAGE, “English”)) AND (LIMIT-TO (DOCTYPE, “ar”))
PubMed	(pneumonia[Title/Abstract]) AND (“deep learning”[Title/Abstract] OR “machine learning”[Title/Abstract] OR “artificial intelligence”[Title/Abstract] OR CNN[Title/Abstract] OR transformer[Title/Abstract] OR “neural network*”[Title/Abstract]) AND (“chest x-ray”[Title/Abstract] OR radiograph[Title/Abstract] OR CT[Title/Abstract] OR imaging[Title/Abstract] OR clinical[Title/Abstract] OR laboratory[Title/Abstract] OR EHR[Title/Abstract] OR biomarker[Title/Abstract] OR “vital signs”[Title/Abstract] OR audio[Title/Abstract] OR “lung sound”[Title/Abstract] OR auscultation[Title/Abstract] OR multimodal[Title/Abstract] OR “data fusion”[Title/Abstract]) AND (“2023/01/01”[Date - Publication]: “2026/07/31”[Date - Publication]) AND english[Language] AND journal article[Publication Type]
Web of science	TS = (pneumonia) AND TS = (“deep learning” OR “machine learning” OR “artificial intelligence” OR CNN OR transformer OR “neural network*”) AND TS = (“chest x-ray” OR radiograph OR CT OR imaging OR clinical OR laboratory OR EHR OR biomarker OR “vital signs” OR audio OR “lung sound” OR auscultation OR multimodal OR “data fusion”) AND DOP = (2023-01-01/2026-07-31)
IEEE Xplore	(“All Metadata”:pneumonia) AND (“All Metadata”:“deep learning” OR “All Metadata”:“machine learning” OR “All Metadata”:“artificial intelligence” OR “All Metadata”:CNN OR “All Metadata”:transformer OR “All Metadata”:“neural network”) AND (“All Metadata”:“chest x-ray” OR “All Metadata”:radiograph OR “All Metadata”:CT OR “All Metadata”:imaging OR “All Metadata”:clinical OR “All Metadata”:laboratory OR “All Metadata”:EHR OR “All Metadata”:biomarker OR “All Metadata”:“vital signs” OR “All Metadata”:audio OR “All Metadata”:“lung sound” OR “All Metadata”:auscultation OR “All Metadata”:multimodal OR “All Metadata”:“data fusion”)

The retrieved records were imported into a reference management software, and duplicate records were removed before screening. The updated search retrieved a total of 6,890 records, including:

- Scopus (*n* = 3,255)
- PubMed (*n* = 1,189)
- IEEE Xplore (*n* = 175)
- Web of Science (*n* = 2,271)

After duplicate removal, 3,799 unique records proceeded to title and abstract screening.

### 2.2 Inclusion and exclusion criteria

Study selection was based on the following pre-established inclusion and exclusion criteria using the PICOS framework:

Population (P): People diagnosed with or suspected of having pneumonia.

Intervention (I): Artificial intelligence techniques, including machine learning and deep learning.

Comparator (C): Existing AI-based methods used for comparison in the included studies, where applicable.

Outcomes (O): AI-based performance metrics (e.g., accuracy, sensitivity, specificity, AUC, and F1-score).

Study Design (S): Original research articles.

Articles were eligible if they used artificial intelligence, machine learning, or deep learning for pneumonia-related detection, diagnosis, classification, segmentation, risk prediction, or prognosis. They were included when they addressed one or more aspects related to the objectives of this review. These included healthcare data modalities, AI architectures, multimodal integration or fusion, explainability, benchmark datasets, and emerging AI approaches for pneumonia diagnosis. The studies that involved the use of medical imaging data, clinical or laboratory data, respiratory audio recordings, or multimodal data analysis were included. Only peer-reviewed journal articles written in English were considered. The review also excluded studies that did not use artificial intelligence, machine learning or deep learning approaches or that lacked an emphasis on pneumonia. Reviews, editorials, commentaries, and surveys were among the additional articles that were not included. Duplicate studies and overlapping datasets, and non-human or simulation-based research were not considered. As a result, 76 studies formed the qualitative synthesis. Multimodal learning was the main focus of the review, while studies based on individual healthcare data modalities also contributed to the analysis. These provided information on diagnostic performance, AI architectures, benchmark datasets, and the limitations of individual modalities. This helped place the development of multimodal approaches in context. Emerging AI methods also received attention when relevant to pneumonia diagnosis. Their possible role in multimodal clinical AI was examined in areas such as interpretability, privacy, scalability, and generalization.

### 2.3 Study selection

The PRISMA 2020 model was followed in this study selection process, which included the identification, screening, and eligibility assessment stages as illustrated in Figure 2. After removing duplicates, 3,799 articles were left for the title and abstract screening stage.Two reviewers independently screened the titles and abstracts of the retrieved records according to the predefined inclusion and exclusion criteria. Articles considered potentially eligible were then reviewed in full text by both reviewers. Any discrepancies between the reviewers were discussed and resolved through consensus. Of these, 2,802 were rejected because they were not pertinent to the study’s goals. All 997 articles’ full texts were successfully obtained once they were assessed for eligibility. During the full text screening stage, 921 articles were eliminated from the analysis based on predetermined inclusion criteria. Consequently, 76 studies qualified for the qualitative synthesis. Although multimodal learning was the main focus of this review, studies using individual healthcare data modalities also formed part of the analysis. They provided information on diagnostic performance, AI architectures, benchmark datasets, and the limitations of individual modalities. These findings helped place the development of multimodal approaches in context. Emerging AI methods were examined when relevant to pneumonia diagnosis. Their potential role in multimodal clinical AI was explored in areas such as interpretability, privacy, scalability, and generalization.

**Figure 2:**
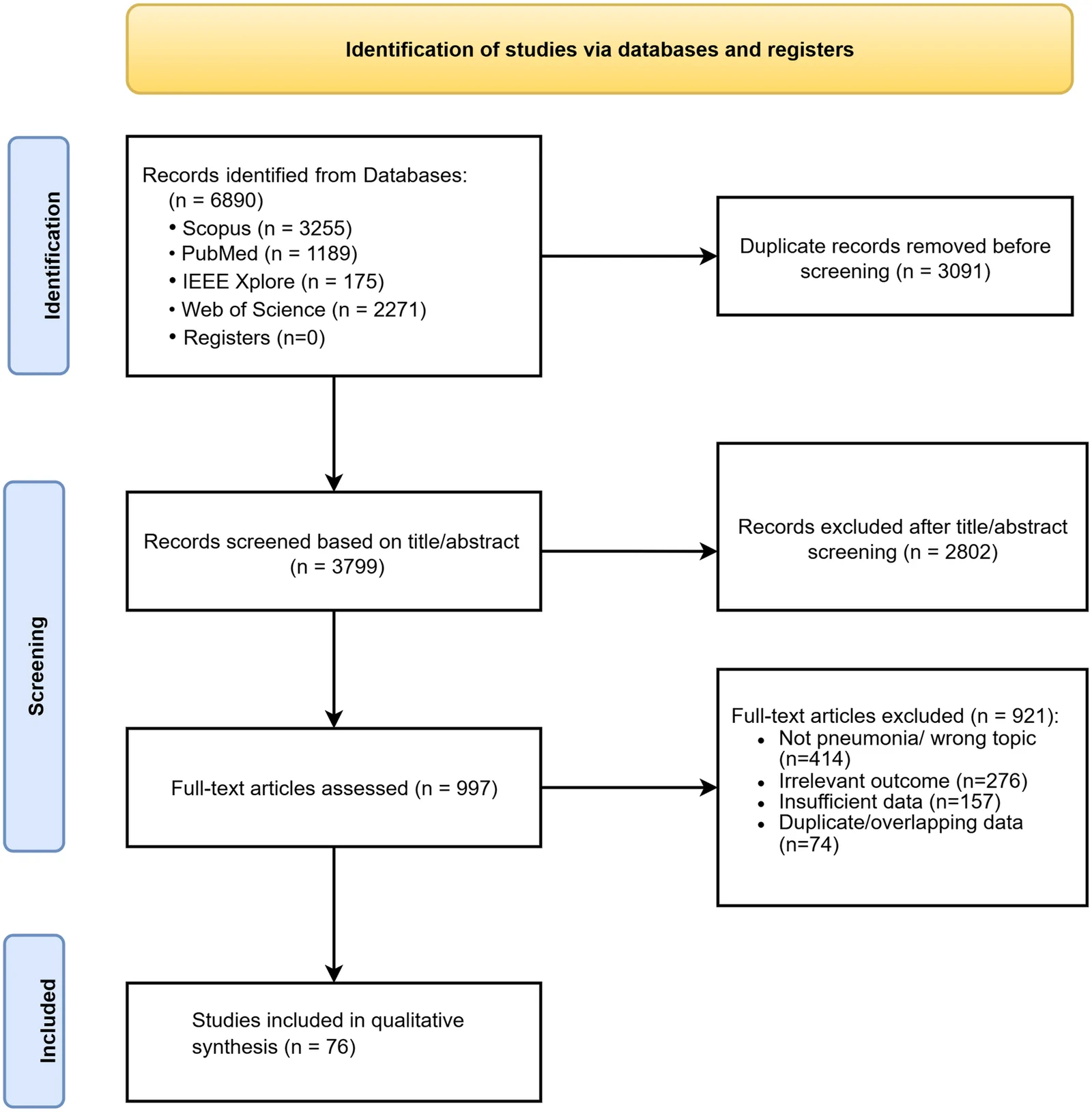
PRISMA 2020 flow diagram illustrating the study identification, screening, eligibility assessment, and final inclusion process.

### 2.4 Data extraction and study evaluation

Data extraction was carried out utilizing a systematic procedure that was prepared before data analysis to ensure consistency and reliability in the collection of data across all the selected investigations. The following were noted for every study:

- Study characteristics include authorship, publication year, journal, and study goal.
- Dataset characteristics include the number of participants used, the source of the data, and the modality of the data (chest X-rays, CT scans, clinical data, respiratory audio, multimodal).
- Methodological characteristics include the sort of fusion employed in multimodal research and the network architectures (CNN, RNN, transformers, hybrid models).
- Evaluation methodology: research design (train-test split, cross-validation, external validation) and evaluation metrics such as accuracy, sensitivity, specificity, AUC, and F1-score.
- Results: key findings and analysis outcomes.

For structured comparative analysis, studies were further categorized into unimodal (imaging, clinical or audio) and multimodal groups.

### 2.5 Methodological quality assessment

The included studies differed in their data modalities, deep learning architectures, validation strategies, and evaluation metrics. Therefore, a structured qualitative assessment was carried out to examine methodological quality and reporting completeness. Five criteria were used: (1) sample size and dataset description quality, (2) model development and validation, (3) performance measures, (4) reproducibility, and (5) practical applicability. Each criterion was rated as inadequate, partial, or adequate. Dataset reporting considered the completeness of information on sample size, dataset source, and relevant dataset characteristics. Model development and validation focused on the clarity of the validation procedure and whether internal or independent/external validation was used. Performance measures were evaluated according to the appropriateness and completeness of the reported diagnostic or model evaluation metrics. Reproducibility focused on the availability of implementation details, code, data, or other resources that could support replication. Practical applicability considered the extent to which clinical or real-world relevance was discussed or evaluated. The ratings of inadequate, partial, and adequate represented insufficient, partially satisfactory, and sufficiently comprehensive fulfilment of the criteria, respectively. The assessment helped identify methodological strengths and limitations among the studies selected for analysis.

### 2.6 Data synthesis

A qualitative synthesis approach has been used because the selected studies varied notably in terms of healthcare data modalities, deep learning models employed, multimodal fusion approaches, validation, and evaluation metrics. The suggested framework that allowed for their comparative analysis was used to analyse the investigations. The framework also incorporated explainability approaches and benchmark datasets, enabling the identification of current research trends and existing gaps in the literature.

## 3 Results

This section presents the findings of the included studies. The discussion covers healthcare data modalities, deep learning architectures, multimodal fusion strategies, explainable AI, benchmark datasets, and evaluation metrics together with their clinical relevance.

### 3.1 Characteristics of included studies

The qualitative synthesis included 76 studies that were carried out between 2023 and 2026. The majority focused on using images, particularly chest X-rays, to diagnose pneumonia. Other studies also included clinical or laboratory data, respiratory sounds, and CT scans. Multimodal techniques, which combine multiple types of input data to improve diagnostic performance, are becoming increasingly common. In addition to several multimodal fusion techniques, a number of deep learning techniques, including CNNs, vision transformers, and hybrid models, have been used. Additionally, all models have been assessed using assessment metrics and publicly available benchmark datasets.

The studies covered several clinical applications, such as pneumonia diagnosis and classification, lesion detection or localization, risk prediction, severity and prognosis assessment, mortality prediction, and treatment-related prediction. These applications were treated as separate clinical tasks because they differ in their objectives, outcome definitions, and evaluation criteria. The analysis first considered the healthcare data modalities and AI architectures used in the included studies. Multimodal fusion and explainability methods were then reviewed. Unimodal studies were retained to provide modality-specific baselines and identify limitations that support the use of multimodal approaches. Emerging AI technologies were also included as complementary approaches. Their potential role in addressing challenges such as limited data, heterogeneous sources, privacy, missing modalities, and generalization was examined. The main findings are presented and discussed in the following sections. Table 2 displays representative samples of the studies reviewed in this review. This illustrates the variety of types of data used in artificial intelligence algorithms to diagnose pneumonia. These models make use of clinical and demographic data, respiratory sound data, and medical imaging data.

**Table 2:** Representative AI-based pneumonia detection studies across different diagnostic modalities.

Related work	Dataset & sample size	Model	Performance metrics	Limitations
Chest X-ray (CXR) studies				
(21)	Pediatric CXR (Kermany): 5,856 (1,583 normal; 4,273 pneumonia)	Attention ResNet with spatial/channel attention, focal loss	Accuracy 98% (baseline ∼96%)	Single dataset; binary only; limited multi-center validation
(22)	7 datasets: 33,920 (COVID-19, pneumonia, healthy)	ResNet18 + Vision Transformer with multi-head attention	Binary 99.3%; 3-class 95.2%; 4-class 90.0%	2D CXR only; lower multi-class; limited interpretability
(23)	Guangzhou CXR: 5,856; COVID19_CXR: 6,939	SAGCN with k-hop graph and attention fusion	Accuracy 98.34%, 97.22%	Limited validation; high computational cost
(24)	PneumoniaMNIST:5,856 Guangzhou:5856 NIH:5941 PediCXR:9,125	Mixed-scale dynamic attention transformer (LLM-based)	Accuracy 96%–98%; AUC up to 0.990	High computational complexity; limited interpretability; needs validation
(25)	NIH CXR14: 112,120 images; 880 annotated	Multi-task CNN (classification + localisation)	AUC 0.918; IoU 0.855	Needs expert annotations; limited labeled samples
(26)	Kaggle CXR: 5,856	Dual-CNN (VGG16 + ResNet) + ML classifiers (SVM, RF)	SVM Accuracy 98.8%, F1 0.98; RF Accuracy 95.8%	Sensitive to image quality; binary only; no explainability
(27)	Pediatric CXR: 5,856 (normal, viral, bacterial)	DAViT (ViT + CNN) with Grad-CAM	Accuracy 96%, F1 97%, AUC 0.96	Single dataset; high computational cost
(28)	ChestXRay2017: 5,856; RSNA: 26,684	PKA2-Net (ResNet + spatial attention)	Accuracy 97.63%, Sensitivity 98.72%	Requires tuning; limited backbone flexibility
(29)	Two CXR datasets: 15,761 (pneumonia/non)	AGPDA with RADB and RFRB	Mean Acc 90.03%	Domain shift issues; limited datasets
CT studies				
(3)	RIDER CT (size not reported)	MGNC (GoogleNet CNN) with IINLCA	Sensitivity 98%, Specificity 98%, Accuracy 98%	Dataset size unclear; no external validation
(4)	Kaggle CT (1,196); RSPHC (494 annotated)	UNet++ segmentation model	Dice 0.994; IoU 0.989	Segmentation only; limited annotations
(5)	Multiple CT datasets: 90,000 slices	Bayesian DenseNet121 + detection models	Accuracy 94.61%; Dice 0.9003	Overfitting risk; annotation variability
(30)	Multi-center CT dataset (train/val/test)	Pneumonia-Plus (HarDNet)	AUC up to 0.934	Limited geography; no multimodal data
(31)	120 COVID-19 CT scans (TCIA)	LungQuant (CNN + U-Net pipeline)	AUC up to 0.98	Small dataset; limited generalization
Clinical/EHR studies				
(32)	EDHS 2016: 2,035 children	Random Forest (best model)	AUC 91.8%, Accuracy 83.3%	Survey-based; limited clinical validation
(33)	MIMIC-III: 3,658 patients	Self-attention CNN with BioWordVec	AUC 99.95%	Single dataset; limited external validation
(34)	Clinical samples (*n* = 50)	CRISPR-Cas12a + DL optimization	>95% concordance	Small sample size
Respiratory audio studies				
(18)	ICBHI + Coswara datasets (>5,000 recordings)	CNN–RNN (LSTM)	Accuracy 92.8%, AUC 0.95	Limited datasets; multi-disease focus
(35)	Kaggle audio: 336 (augmented to 840)	CNN feature fusion framework	Accuracy 96.03%	Small dataset; oversampling bias
(36)	CCAP-LSD: 198 children; 1,188 recordings	BPAM (VGGish + BiGRU)	Sensitivity and Specificity >92%	Limited dataset; pediatric only
(37)	173 children; 491 cough events	SincNet-BiGRU + MLP	Accuracy 76.33%	Small dataset; no external validation

### 3.2 Healthcare data modalities for pneumonia diagnosis

The diagnosis of community-acquired pneumonia is typically made based on the patient’s symptoms and chest imaging; nonetheless, laboratory and microbiological testing may be required to confirm the diagnosis (38). Due to recent studies, multimodal AI models that incorporate imaging data together with other types of data are becoming popular because physicians typically employ data from various sources for interpretation. Compared to single-modality-based models, this method may offer precise diagnostics.

Medical imaging data remains the primary basis for AI-based pneumonia diagnosis. The most popular imaging methods are chest X-rays (CXR) and Computerized Tomography (CT) which offer a great means of identifying lung issues such as consolidation and ground-glass opacities (30, 39). Medical imaging data analysis is dominated by Convolutional Neural Network (CNN)-based methods. Because they offer superior image feature extraction and classification, transformer-based methods have achieved excellent results in recent years (22, 24, 27). These imaging applications are linked to several clinical tasks. Classification determines whether pneumonia is present or assigns a disease category, whereas lesion detection and localization identify abnormal regions within the image. Therefore, performance across these tasks should be interpreted separately. However, there is still ample room for improvement in AI models for medical imaging. Many studies lack external validation, rely on small or biased datasets, and generalise poorly across data from various healthcare institutions (23, 25, 26).

The main data modalities used to diagnose pneumonia are illustrated in Figure 3. These data modalities fall into four categories: respiratory signals, clinical data, medical imaging data, and multimodal data fusion. Lung Ultrasound (LUS), CT, and CXR are among the medical imaging data. Vital signs, lab markers, demographics, and Electronic Health Records (EHRs) are common instances of clinical data (40, 41). Lung noises, coughing sounds, and breath sounds are examples of respiratory signals (42). Combining at least two of these data modalities results in multimodal data fusion.

**Figure 3:**
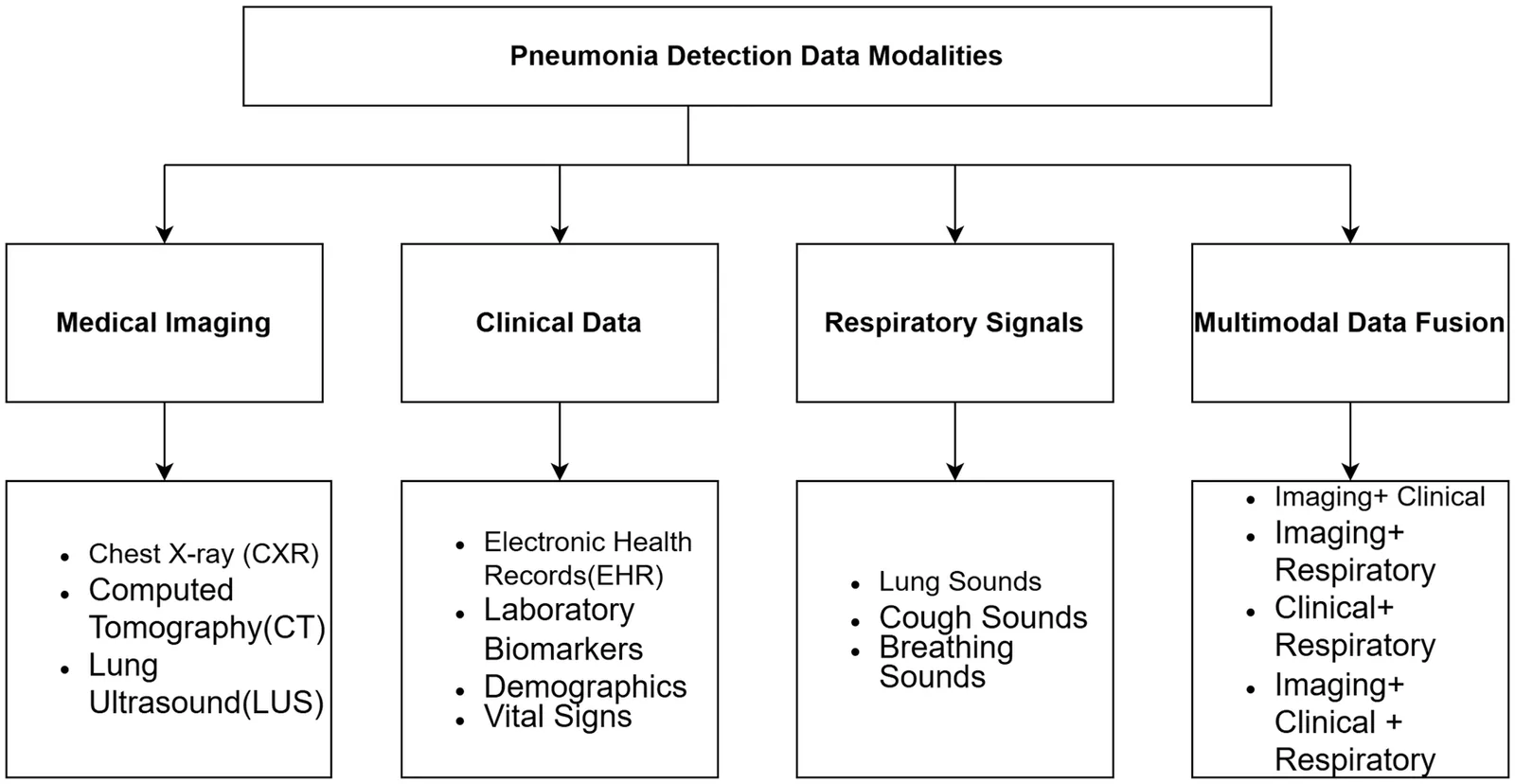
Overview of the major healthcare data modalities used for pneumonia detection, including medical imaging, clinical data, respiratory signals, and multimodal data fusion.

### 3.3 Deep learning architectures

Deep learning was the most common architecture utilized in all of the reviewed studies. While transformer networks were common for analysing long-range image data, Convolutional Neural Network (CNN) architectures were the most often utilized type in chest X-rays. To incorporate both imaging and clinical data, several architectures were tested, including hybrid architectures, graph neural networks, recurrent neural networks, and multimodal fusion architectures. The chosen architecture was often determined by the diagnostic goal and the data utilized.

#### 3.3.1 CNN and transfer learning

CNN architectures are the most commonly used models for diagnosing pneumonia from chest X-ray and CT images among the analysed studies. CNNs are widely used because they can identify spatial patterns in medical images. They have been used in numerous medical applications, including pneumonia classification, lesion detection, and abnormality localization. Differentiating these applications is necessary for assessing the network’s efficacy. Classification evaluates disease-level predictions, whereas detection and localization focus on identifying abnormalities and their location within an image. ResNet, DenseNet, EfficientNet, GoogLeNet, and U-Net are popular CNN designs that have demonstrated great efficiency in various diagnostic situations (3, 30, 39). In certain experiments, the classification challenge was solved by combining conventional machine learning classifiers with pretrained CNN feature extractors (43). Additionally, some studies used attention mechanisms that allow CNN models to exclude irrelevant features from the image and focus on relevant regions of the lungs (21, 28). Among recent unimodal imaging methods, Barman and Giri (44) proposed eRes-NET, an ensemble of residual CNNs for pneumonia detection. The model combines localized chest X-ray feature extraction with image enhancement techniques. This approach illustrates the continued development of optimized CNN-based methods alongside emerging multimodal architectures.

There are several challenges that need to be resolved. The models will perform differently depending on the type of imaging modality and the characteristics of various patients at various healthcare settings. Furthermore, there is a lack of external validation and high reliance on large data sets.

In spite of their strong performance, CNN models have some drawbacks. Convolutional operations primarily capture local spatial features and may not adequately model long-range dependencies within medical images. In addition, the models’ generalizability may be limited by differences in model performance among patient populations, imaging protocols, and annotation practices. External validation was also limited, with only a few studies evaluating CNN models on independent datasets. The results indicate that further validation in different clinical settings is required to enhance the overall applicability and robustness of CNN-based pneumonia diagnosis.

#### 3.3.2 Transformer-based architectures

Transformer-based architectures have recently gained popularity as a substitute for conventional Convolutional Neural Network (CNN) models in the diagnosis of pneumonia. Vision Transformers (ViTs) and hybrid CNN-Transformer models are able to retain significant local features while learning relationships between distant portions of an image. They are also suitable for chest imaging classification and segmentation applications (6, 7, 27). Further, some studies have combined CNN backbones with transformer modules to improve diagnostic performance, especially when analysing complex imaging datasets.

More recently, Abdullah et al. (45) proposed HybridCATNet, an explainable hybrid CNN–Transformer framework for pneumonia detection from chest X-rays. The model uses raw, lung-masked, and edge-enhanced image representations through early multi-channel fusion. CNNs extract local features, whereas Transformers capture global representations, with spatial–channel attention used during feature processing. Grad-CAM and SHAP were included for explainability, while Bayesian dropout was used to estimate uncertainty. HybridCATNet was also tested on independent external datasets without fine-tuning. This work reflects recent efforts to develop pneumonia detection models that provide explanations, account for prediction uncertainty, and maintain performance across different datasets.

Despite certain benefits, transformer-based models still face a number of difficulties. They utilize a self-attention system that is computationally complex. Large, well-annotated datasets are also usually needed to train these models. As a result, their application might be restricted in healthcare environments with limited resources. According to certain research, transformer-based topologies were effective at identifying intricate pneumonia patterns. However, large clinical usage is still restricted by their high computational demands.

#### 3.3.3 AI models for clinical and multimodal healthcare data

Beyond medical imaging, deep learning has been applied to a wide range of healthcare data, including clinical information, EHRs, laboratory test results, respiratory sounds, and molecular diagnostic data. Such studies show the strengths and limitations of each data source. It also provides a basis for combining different types of information in multimodal systems. Using clinical and demographic data, a number of studies have examined the use of machine learning techniques such as Random Forest, Support Vector Machine, Logistic Regression, and Decision Tree to assess pneumonia risk (32, 46). Several Natural Language Processing (NLP) algorithms have been put forth concurrently to examine clinical narratives and EHRs. It has been reported that pneumonia-related information may be extracted from clinical writing using self-attention, convolutional neural networks, and biological language embeddings (33). Hybrid CNN-RNN architectures can learn both spatial and temporal information from acoustical inputs, they have produced excellent results in respiratory sound analysis (35, 36, 47). Additionally, deep learning-assisted CRISPR-based molecular diagnostic methods have made it easier to identify diseases using molecular markers (34).

The aforementioned literature reviews point out how imaging combined with clinical, laboratory, respiratory, and genetic data can yield useful information on disease prognosis and severity evaluation. The reviewed studies show that multimodal approaches depend on the quality and availability of the input data. Incomplete and varied data were also important issues in developing these models.

#### 3.3.4 Synthesis of deep learning architectures

It is evident from the reviewed literature that CNNs continue to dominate medical image analysis because of their effectiveness and exceptional outcomes in typical medical imaging data. Conversely, transformer networks are more likely to be chosen when it is helpful to model long-range dependencies within the image. Apart from imaging, there are several instances of AI algorithms that employ clinical and other healthcare-related data to carry out tasks like risk assessment or prognosis prediction. The development of numerous hybrid and multimodal techniques that utilize diverse network types based on data attributes has been prompted by this tendency. The multimodal fusion techniques that have been used to accomplish that goal are discussed in the next section.

### 3.4 Multimodal fusion strategies

Multimodal fusion strategies combine complementary information from different healthcare data sources, such as CT and chest X-ray images, laboratory results, clinical or textual data, respiratory data, and molecular data. In this review, these approaches were grouped into three main categories: early fusion (feature-level integration), late fusion (decision-level integration), and hybrid fusion. Attention mechanisms were treated as complementary techniques rather than a separate fusion level. They can be incorporated into different fusion approaches to determine the relative importance of features or data modalities. Hybrid fusion combines information at two or more stages of the modelling process, whereas attention-based methods assign greater or lesser weight to features or modalities during integration. Thus, a multimodal model can use both hybrid fusion and attention mechanisms.

#### 3.4.1 Early fusion (feature-level concatenation)

Prior to classification, early fusion integrates data from multiple sources. A single representation is created by combining the features from lab reports, clinical data, and medical imaging. This ensures that the model can examine all the data collectively and discover connections between distinct modalities that could have been overlooked if the various sources were handled independently.

When each patient has numerous data modalities that can be precisely synchronized, early fusion performs best. Wang et al. (48) demonstrated this by diagnosing children community-acquired pneumonia using laboratory biomarkers, clinical data, and chest X-rays. Bi-GRU was used to represent the text data, ResNet-50 was used to extract image features, and deep neural networks were used to handle laboratory data. The suggested fused feature representation performed with 0.979 AUC and 94.2% accuracy. This conclusion is also supported by the findings of CheXMed (49), which merged data from electronic medical records with features from chest X-rays. Overall, the reviewed studies suggest that combining imaging and clinical data can provide complementary information and may improve patient representation and pneumonia detection performance in specific settings.

Early fusion has a number of serious disadvantages, even though it is a suitable option when all the multimodal data are available. High dimensionality is often achieved by merging characteristics from various data sources, which may result in overfitting and demand additional processing resources. Early fusion cannot be used without data because its performance depends on the availability of all modalities. This could make it difficult for the model to capture the relationships between various data modalities.

Early fusion is typically a simple and efficient method of combining health care data with additional information when all the modalities are available. The technique allows the model to use data from several sources by mapping the input to a common feature space. Additionally, the high dimensional data representation that results from the integration of numerous features raises computing costs and may cause overfitting.

#### 3.4.2 Late fusion (decision-level integration)

Late fusion, also known as decision-level integration, combines the predictions generated by models trained on different data modalities. Whether the data is an EHR, a laboratory result, or a medical image, each model extracts the properties that correlate to its type of data. Instead of merging the raw data or extracted features, the individual predictions are integrated to produce the final diagnosis. Common late fusion methods include weighted average, majority vote, and meta-learning. In contrast to early fusion, this method is resilient if some modalities are either completely or partially unavailable. These are a few reasons why late fusion would be suitable for real-world medical applications.

Several researchers explored late fusion methods for pneumonia diagnosis. Using transfer learning-based CNN models such as MobileNetV2, ResNet, and DenseNet, a late-fusion-based multimodal framework extracts features from CT, chest X-ray, and lung ultrasound images. These results are then combined using Support Vector Machines at the decision level. Through iterative weight pruning, the suggested method has improved computation efficiency and achieved accuracies higher than 99% (13). Similarly, features from chest X-ray and CT images are incorporated into the Loss-Attentional Physics-Informed Neural Network (LAPINN) model, which is optimised with the Lotus Effect Algorithm (LEA) to diagnose pneumonia. The LAPINN model attained 99.5% accuracy, 98.5% recall, and 97.2% precision (50). Despite its flexibility, late fusion still has several disadvantages. First, the interactions between complementary features cannot be directly incorporated into the learning process because each modality is handled independently. Second, the different weights of each modality might not be taken into consideration by conventional methods of combining the classifiers, including average or majority voting. As a result, the model may not fully utilize the available multimodal information, which can affect its diagnostic performance.

Overall, existing studies show that late fusion is a highly adaptable and modular method for integrating diverse datasets in the healthcare field. Because all data modalities are not always available, late fusion allows each modality to be processed independently, making it suitable for healthcare applications. However, the independent processing of modalities limits its ability to learn complex interactions between different data sources. To overcome this limitation, recent research has focused on developing hybrid and attention-based fusion strategies that can capture deeper relationships among multiple healthcare modalities.

#### 3.4.3 Hybrid and attention-based fusion

Hybrid fusion models combine information from different fusion levels, such as feature-level and decision-level representations. Hybrid fusion and attention-based fusion are related, but they are not the same. Hybrid fusion merges two or more fusion levels or strategies, whereas attention-based fusion uses attention mechanisms to assign different weights to features or modalities during integration. A hybrid model can also use attention mechanisms. Thus, the two approaches may overlap and should not be treated as mutually exclusive categories. Hybrid models can combine intermediate features from multiple healthcare sources before producing the final output, while attention mechanisms can help emphasize the most relevant information during integration. Compared with early and late fusion models, hybrid fusion models are better at capturing intricate relationships among different data sources. Better diagnosis of COVID-19, bacterial, and viral pneumonia has been linked to better depiction of interactions between data sources.

Hong et al. (15) used plasma proteomics and chest CT image data to develop Image-Expression Axes associated with disease severity, treatment effectiveness, and mortality risk. PneumoFusion-Net (16) and the suggested method from Xing et al. (17), which made use of imaging, clinical, laboratory, and genetic data, both employed the same multimodal approach. The multimodal integration considerably improved the pneumonia categorization with accuracy exceeding 95% in each example.

Recent works show continued progress in multimodal approaches to pneumonia diagnosis. Hofmeister et al. (51) combined chest radiographic data with clinical and biological variables, showing the value of using imaging together with patient-level data. Fu et al. (52) introduced STAR-ViT, which uses CT and chest X-ray data for cross-modal representation learning and adversarial alignment in pediatric pneumonia pathogen classification. Together, these studies show a growing interest in combining clinical data from different sources with cross-modal learning for pneumonia diagnosis.

According to these evaluated articles, the multimodal pneumonia diagnosis methodology now employs hybrid and attention-based fusion procedures. This is accomplished by integrating data from several modalities, such as imaging, clinical, laboratory, and molecular data. The acquired results clearly suggest that they are superior to the conventional methods for early and late fusion. Nevertheless, their application in clinical settings is still hampered by several issues, such as high computational costs, the need for a large multimodal dataset, parameter tuning, and interpretability challenges.

Collectively, the reviewed studies emphasise how fusion strategies have evolved from traditional methods to complex approaches incorporating hybrid and attention mechanisms. As previously discussed, late fusion offers greater flexibility when working with multimodal data in healthcare data, although early fusion is a very practical method of doing so. However, when it comes to applying adaptive feature fusion to learn the links between different modalities, hybrid and attention mechanisms are effective. Therefore, these approaches are used frequently in multimodal systems for pneumonia diagnosis because they improve robustness, diagnostic accuracy, and clinical applicability.

### 3.5 Explainable AI in multimodal models

In addition to strong diagnostic accuracy, the implementation of multimodal Artificial Intelligence (AI) in clinical practice entails an understandable decision-making process. Although multimodal deep learning architectures are efficient in merging imaging, clinical, and laboratory data, the complex structure of such systems interprets prediction outcomes problematic. In addition to restricting the dependability and acceptance of multimodal AI by physicians, inadequate understandability offers additional hurdles for the regulation of such solutions. To overcome the above challenges, the usage of Explainable AI (XAI) becomes a crucial feature of medical AI technologies.

XAI makes the process of using AI to diagnose pneumonia transparent by giving doctors insight into how AI models generate their outputs and predictions. As a result, using AI to diagnose pneumonia improves its interpretability. The importance of XAI in such applications has recently been proven, as there are progressively instances of employing AI models to diagnose diseases using images and other multimodal data (53).

#### 3.5.1 Visual explanation techniques

Visual explanation methods help clinicians detect the specific image areas that affect model predictions. One of the most widely used methods for interpreting CNN-based pneumonia prediction models is Gradient-weighted Class Activation Mapping (Grad-CAM), which produces heat maps displaying the regions of interest associated with lung pathology, such as consolidation, infiltration, and ground-glass opacities. Similarly, Class Activation Mapping (CAM) was applied to pediatric chest X-ray images to identify pneumonia-related areas. A visual confirmation of the model’s predictions was provided by the detected regions, which appeared to correspond with radiologists’ observations (54). Several advanced variants of Grad-CAM have also been developed to improve the quality of explanations. While Score-CAM may produce visual explanations without using gradient information, Grad-CAM++ can help identify various regions of interest. Principal component analysis is used in another version, Eigen-CAM, to identify the model’s most significant characteristics (55). The graphical approaches enhance the explainability of the deep learning models and maintain strong agreement with radiology findings (56). In multimodal systems, visual explanation techniques can also provide insights into how imaging characteristics are combined with clinical information to support diagnostic predictions (57, 58).

#### 3.5.2 Explainability for clinical and multimodal data

Models trained using multimodal healthcare data and structured clinical data have also been tested for explainability. While Huang et al. (59) also used LIME for local explanation of individual predictions, SHAP was used in a study employing structured clinical factors to identify which variables most significantly affected the model predictions (60). Similarly, Li et al. (61) used SHAP to identify significant indicators of mortality risk, including Body mass index, albumin, age, and Aspartate Aminotransferase (AST). In order to recognize the area of the image associated with diseases as well as the contribution of CT images, clinical text, lab values, and radiology reports, Wang et al. (16) used Grad-CAM and the attention approach in multimodal modelling. By comprehending how clinical factors and other data modalities affect the AI model’s decision-making, explainability techniques have collectively improved the models’ interpretability.

#### 3.5.3 Clinical trust

The formulation of understandable explanations in addition to the model predictions can increase clinical confidence in the application of AI-supported pneumonia detection. The reviewed studies demonstrated that explainability techniques improved the degree of explainability of the prediction models by assisting physicians in understanding the components of images and clinical factors that are involved in making predictions (59, 61, 62). Ayub et al. (63) used Monte Carlo Dropout to estimate uncertainty and Grad-CAM to explain CXR-based pneumonia screening results. The work considers not only the classification result but also the reliability and interpretation of the model’s predictions. These additions may support safer use of AI systems in clinical decision-making. Still, post-hoc explainability techniques were employed in the majority of the examined research.

### 3.6 Evaluation metrics and clinical relevance

The performance of AI models for pneumonia diagnosis is assessed using several evaluation metrics, including accuracy, sensitivity, specificity, precision, F1-score, and the Area Under the receiver operating characteristic Curve (AUC) (64). Every metric describes specific aspects of the model performance. The percentage of accurate predictions is displayed by accuracy. The model’s ability to detect pneumonia patients is referred to as sensitivity. This is an especially important requirement since failing to do so could cause treatment to be delayed. The algorithms’ ability to accurately classify individuals who do not have pneumonia is referred to as specificity. When working with an unbalanced data set, precision and F1-score can be used to provide a fair evaluation of positive predictions.

The AUC measures how well a model differentiates between pneumonia and non-pneumonia cases across different decision thresholds. The Dice Similarity Coefficient (DSC) and Intersection over Union (IoU), which measure the degree of overlap between the predicted and reference lung areas, were used to evaluate image segmentation performance (57). The evaluation metrics were interpreted based on the clinical task. Accuracy, sensitivity, specificity, precision, F1-score, and AUC were mainly used for diagnostic and classification tasks. For segmentation and localization, Dice Similarity Coefficient (DSC) and Intersection over Union (IoU) were used. Risk prediction, prognosis, severity assessment, and mortality prediction may require different outcome definitions and task-specific measures. Therefore, performance values across different clinical applications were not considered directly comparable, even when the same metric was reported. The applicability of AI models in clinical practice depends not only on performance metrics but also on their robustness, external validation and consistency across different healthcare settings. Several studies reported that deep learning models exhibited diagnostic accuracy on an equal basis with or higher than radiologists. Both the performance of the modality itself and the added value of data fusion should be considered when evaluating multimodal approaches (65).

### 3.7 Benchmark datasets

Large annotated datasets are required for the development of deep learning models for pneumonia detection. Benchmark public datasets are a common resource that may be utilized for performance-based training, validation, and comparison of different methodologies. Such datasets have made it easier to develop and evaluate new methods. These datasets enable researchers to examine different approaches under comparable experimental conditions. Table 3 presents the benchmark datasets used in the reviewed studies, along with their data modalities, sample sizes, applications, and representative research works.

**Table 3:** Representative public and multimodal datasets for respiratory disease and pneumonia diagnosis in AI research.

Dataset	Modality/type	Size	Annotations & use	Limitations	References
RSNA pneumonia detection	CXR (imaging)	∼30 K	Bounding boxes; detection, localization	Limited clinical metadata	(66, 67)
Kaggle CXR pneumonia	CXR (imaging)	∼5.8 K	Binary labels; classification	Pediatric-only; limited generalization	(68)
CheXpert	CXR + reports	224 K	14 labels; multi-label, semi-supervised	Label uncertainty; NLP noise	(69)
MIMIC-CXR	CXR + text	377 K	NLP labels; multimodal learning, report generation	Noisy labels; report ambiguity	(70)
ICBHI 2017	Audio	∼920	Crackles/wheezes; classification	Limited labels	(71, 72)
Coswara	Audio + metadata	>2 K	Symptom labels; screening, multimodal learning	Self-reported labels; noise	(73)
MIMIC-IV + CXR	CXR + EHR + text	377 K + 70 K	ICD + NLP labels; prediction, prognosis	Single-center; missing data	(70, 74, 75)

As shown in Table 3, chest X-ray datasets are the most widely used benchmark resources for pneumonia classification due to their large-scale availability and open access. The Kermany Pediatric Chest X-ray, ChestXRay2017, ChestX-ray14, RSNA Pneumonia Detection Challenge, PneumoniaMNIST, and PediCXR datasets are frequently used. These datasets have enabled researchers to pursue a variety of research objectives, including disease classification, anomaly localisation, and the development of deep learning models using transfer learning techniques (21, 24). Additionally, the development of cutting-edge models for lesion segmentation, illness characterisation, and severity evaluation of bacterial, viral, and COVID-19 pneumonia has been greatly aided by CT imaging data (3, 4, 30).

AI-based methods for detecting and forecasting the risk of pneumonia have been made possible by a variety of structured data sets, including medical imaging data sets. Clinical prediction models can be developed using medical datasets such as MIMIC-III and the Ethiopian Demographic and Health Survey (EDHS), which contain laboratory findings, electronic health records, and demographic information (32, 33). Additionally, automated algorithms for lung sound classification and lung ultrasound evaluation have been developed using breath sound databases such as ICBHI, Coswara, and BEDLUS (18, 19, 36). Molecular diagnostic data have introduced another use of AI technology, namely sets that contain CRISPR-based biosensing data and lab data for particular infections (34).

According to the reviewed studies, the progress of AI-based pneumonia diagnosis has benefited greatly from publicly accessible benchmark datasets. Regretfully, the majority of datasets that are currently accessible are obtained from individual sites and are modality-specific. The absence of multimodal datasets, inconsistent annotation practices, class imbalances, and inadequate external validations are major problems with these datasets. This may have an effect on the AI model’s capacity to generalize to different clinical settings. Larger multi-center multimodal data sets and labelling process consistency are required for future developments. This could aid in the creation of reliable models and AI solutions that can be used in medical settings.

Figure 4 presents the key developments in AI-powered pneumonia diagnosis that were reported in the reviewed studies. The data modalities used in the healthcare field, deep learning architectures, multimodal fusion strategies, explainability methods, challenges in implementing AI technology, and some recent progress in AI are all included in this figure. It also shows how the investigated articles moved from image-focused to multimodal systems.

**Figure 4:**
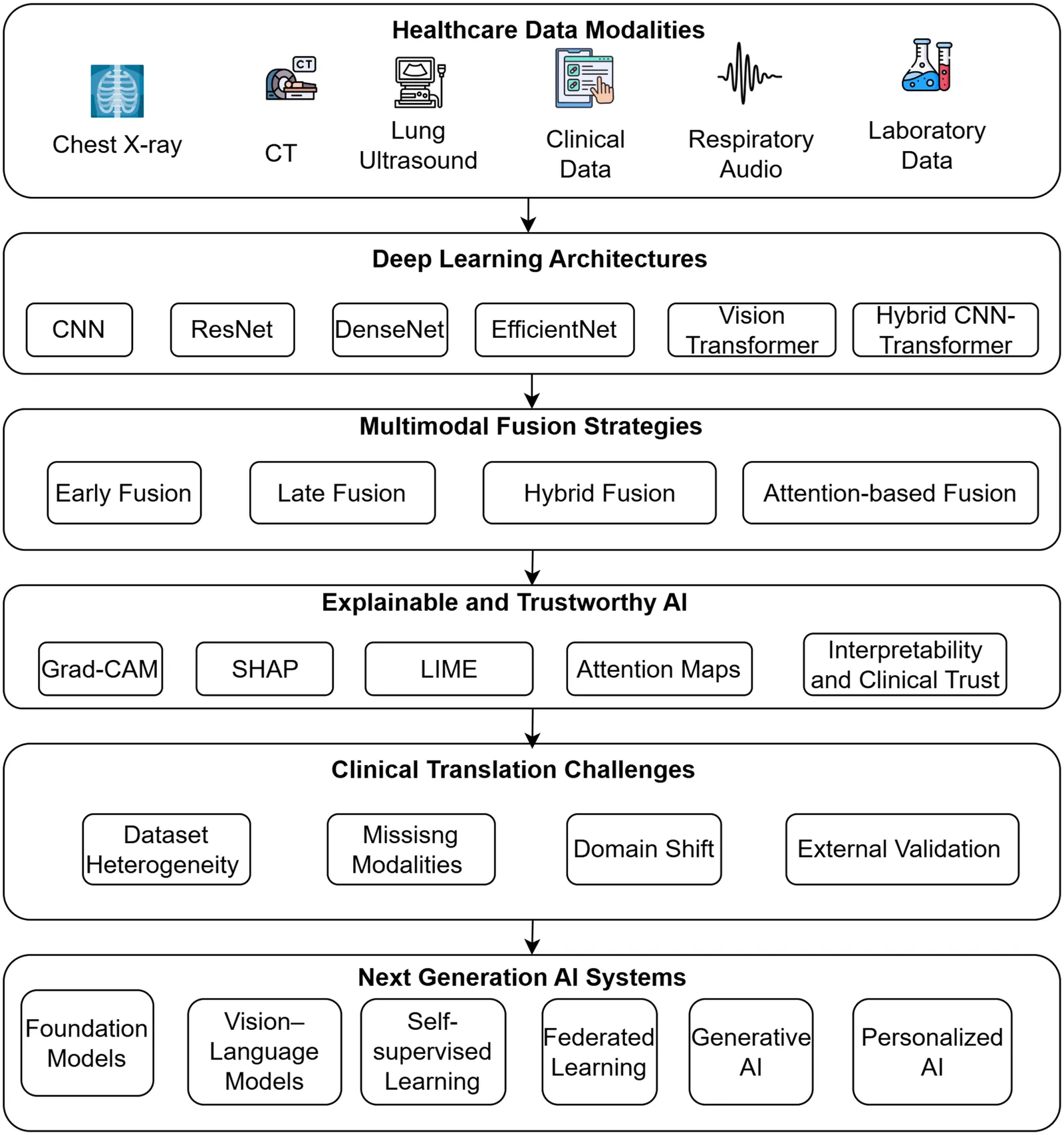
Evolution of AI-based pneumonia diagnosis, highlighting healthcare data modalities, deep learning architectures, multimodal fusion strategies, explainability and trustworthiness, clinical translation challenges, and emerging AI systems.

## 4 Discussion

The findings of the systematic review are discussed in this section that follows in respect to the multimodal AI techniques currently in use for pneumonia diagnosis. The positive and negative aspects of current approaches are examined, as well as difficulties with using them in clinical translation, and outlines important directions for future studies.

### 4.1 Key findings and comparative insights

The included studies indicate a shift from image-only models towards multimodal AI models that fuse multiple healthcare data modalities. The unimodal studies in the review provide a baseline for understanding this progression. The emerging AI approaches discussed later are treated as supporting technologies. They may help in developing more robust and scalable multimodal systems. In any event, chest X-rays and CT scans continue to be the most used imaging modalities for pneumonia diagnosis. However, recent studies indicate that integrating imaging data with clinical data, laboratory results, respiratory sounds, or genomic data may improve the model’s robustness, clinical significance, and diagnostic accuracy (15–17).

The reviewed studies suggest that model selection also depends on the type and availability of input data. CNNs remain useful for standard imaging tasks. Transformer-based and hybrid models can capture relationships across a wider range of image information (27, 30). However, it has been discovered that these models require greater processing power and more training data. This may limit their use in clinical settings with limited resources.

The reviewed fusion strategies show a balance between flexibility and the integration of data from several modalities. If all modalities are present, early fusion enables effective use of complementary information. Because each modality is processed separately, late fusion provides greater flexibility. Hybrid fusion can merge information at different fusion levels, while attention mechanisms can assign different weights to features or modalities during integration. A hybrid model may also incorporate attention mechanisms, so these approaches can overlap but should not be considered equivalent. The availability and compatibility of the data, the processing power, and the intended clinical use should all be taken into consideration when selecting a fusion technique. The reviewed studies suggest that hybrid and attention-based multimodal fusion approaches can help integrate information from multiple healthcare data sources. However, their reported performance cannot be directly compared with unimodal approaches across studies because the datasets, patient populations, clinical tasks, experimental settings, and validation protocols differ. Thus, these approaches should be considered promising rather than consistently superior to unimodal methods.

Recent work points to several emerging trends in pneumonia AI research. Multimodal methods are moving beyond simple feature concatenation by using cross-attention, knowledge-enhanced fusion, and cross-modal representation learning. Semi-supervised learning is another area of interest, particularly for reducing the amount of labeled data required. Uncertainty and explainability are receiving more attention alongside classification accuracy, as they can provide a better view of prediction reliability. However, many of these methods are still evaluated on retrospective or single-center datasets. Independent, multicenter, and clinically representative data are needed to determine how well they perform in real clinical settings. Explainability can help clinicians understand the reasons behind a model’s prediction. It can also show whether the model is focusing on clinically relevant features. However, most explanation methods were evaluated qualitatively. A widely accepted quantitative measure for assessing the reliability of these explanations is still lacking (28, 64).

The majority of the reviewed studies made use of publicly accessible databases that representing individual data modalities. The utilization of multimodal cohorts was not well documented. Different patient groups, annotation approaches, image acquisition, and validation techniques make it challenging to compare different studies. Because some models were tested using retrospective or single-center databases, care must be taken when interpreting the results (8, 25). High diagnostic performance on benchmark datasets does not necessarily mean that a model will work well in clinical practice. Reported results can vary with patient populations, disease prevalence, imaging protocols, data quality, and validation methods. Domain shift and dataset bias may further affect how well a model performs when it is applied to data from other healthcare settings. For this reason, measures such as accuracy, F1-score, and AUC should be interpreted with caution, especially when independent external validation is not available. Table 4 summarizes selected studies and highlights differences in clinical tasks, data modalities, fusion strategies, datasets or cohorts, and reported performance. The performance values in Table 4 are reported based on the clinical objective of each study. They should not be taken as direct comparisons across different tasks. Diagnostic and classification results differ from those for lesion localization, risk prediction, prognosis, mortality prediction, and treatment-related outcomes. Each of these tasks has different clinical endpoints and evaluation requirements.

**Table 4:** Comparative analysis of multimodal pneumonia studies.

Related work	Clinical task	Modalities	Fusion strategy	Dataset/cohort	Key performance metrics
(16)	Pneumonia classification	CT + Clinical text + Lab + Radiology reports	Hybrid fusion with attention mechanism	Hospital cohort; 10,095 CT images	Accuracy: 98.96%, F1-score: 98%
(49)	Pneumonia classification	CXR + Clinical notes/EMR	Early fusion (feature-level)	ChestX-ray14, CheXpert, RSNA, local hospital	Accuracy: 74.6%, F1-score: 74.3%
(48)	Pediatric community-acquired pneumonia diagnosis	CXR + Lab + Clinical text	Early fusion (feature-level concatenation)	Fujian Children’s Hospital; 1,590 patients	Accuracy: 94.2%, AUC: 0.979, F1-score: 0.943
(17)	Prognostic prediction/in-hospital mortality prediction	CT + Clinical + Lab + Microbial	Graph-based multimodal fusion	Single-center; 185 patients + 44 additional validation patients	5-fold CV AUC = 0.9948; additional validation accuracy = 95.45%
(76)	Severity prediction	Clinical + Serum biomarkers + Imaging (CXR & CT)	Feature-level integration with ML ensemble	Prospective cohort; 597 patients	LightGBM AUC = 0.92; SCRPT model AUC > 0.80
(15)	Prognostic prediction/treatment-response prediction	CT + Plasma proteomics	Hybrid fusion (CSRL + PoE + GCN)	CMAISE-COVID19 cohort; 1,979 patients	Mortality: IEA1 OR = 0.52, IEA2 OR = 0.74
(13)	Multiclass classification	CT + CXR + LUS	Late fusion (decision-level ensemble)	Public datasets; 20,793 instances	CT: 99.4%; CXR: 98.9%; LUS: 99.3% accuracy
(77)	Pneumonia prediction	CXR + structured EHR + clinical text + biomedical knowledge	Knowledge-enhanced spatial–temporal attention with feature-level multimodal fusion (K-STAMM)	MIMIC-IV + MIMIC-CXR	Accuracy: 91.2%; AUROC: 0.953; AUPRC: 0.962; F1-score: 0.910
(78)	Checkpoint inhibitor-related pneumonia prediction	CT + EMR text + Physiological test reports	Pairwise cross-attention multimodal fusion (MMCA-Net)	Single-center retrospective cohort; 553 patients (261 CIP + 292 non-CIP)	Accuracy: 87.12%; AUC: 0.8981; F1-score: 85.86%

### 4.2 Clinical translation challenges

The practical implementation of multimodal deep learning for pneumonia diagnosis is currently hampered by a number of issues. The existence of heterogeneous datasets, inadequate data, domain shift, a lack of external validation, and poor explainability are some of these challenges, according to the reviewed articles. Overall, these limitations may reduce model robustness and diagnostic accuracy.

#### 4.2.1 Dataset heterogeneity

The most frequently mentioned issue in the reviewed articles was dataset variability. Using a variety of imaging devices and acquisition techniques, the datasets were collected from different healthcare environments. Due to various annotation techniques, there was variation in both the quality of the images and the quantity of patient data. Most models were developed using publicly accessible data. Even though these datasets have been crucial to the field’s advancement, they do not fully capture all potential differences in the real world. As a result, although the models may function well during development, they will not be able to generalize in real-world situations. Building standardized, well-annotated, multicenter multimodal datasets is therefore an important step toward improving the robustness and clinical reliability of future AI systems (79).

#### 4.2.2 Missing or incomplete modalities

Data from several health care sources is necessary for multimodal AI algorithms to be accurate. However, the patient records are often incomplete in the typical workflow. Certain types of data, such as imaging, lab tests, clinical notes, respiratory sounds, or genetic data, may be lacking (15–17). Many of the multimodal techniques currently in use are based on the assumption that all modalities are accessible. In real-world settings, this assumption is often not valid because one or more data sources may be missing (16, 34). As a result, model performance can decline when incomplete patient information is encountered. Therefore, the development of multimodal learning algorithms capable of handling such issues is imperative.

#### 4.2.3 Domain shift and generalization

Another significant issue affecting the effectiveness of multimodal AI models is domain shift. This issue occurs when the data utilized to create the model differs from the data seen in real-world situations (8, 25). Several factors, including patients, imaging equipment, disease prevalence, and methods, could cause this discrepancy. As a result, the generated models could perform poorly when applied to data from other hospitals, even though they might perform well on a single dataset. Several studies have investigated domain adaptation techniques to improve performance across different datasets (80, 81). While these methods have shown promising results, achieving consistent and reliable performance across geographically diverse clinical environments remains a significant challenge. Consequently, strong results on an internal or benchmark dataset may reflect features of that dataset rather than how well the model works in clinical practice. Patient demographics, disease prevalence, imaging equipment, acquisition protocols, and institutional practices can differ between the development data and those used in practice. These differences may affect model performance after deployment. External validation with data from independent institutions and geographically diverse populations is therefore important for assessing whether the reported performance remains consistent in real-world clinical settings.

#### 4.2.4 Limited external validation

Although several studies have reported good accuracy, they used internal data sets or historical data from a single healthcare organization to validate their model (8, 25, 26). However, a few publications used external data from many healthcare institutions to assess the model’s efficacy (14, 48). It is difficult to assess how effectively the models will function in clinical settings because many of the examined articles had issues with external validation. To verify the generalizability of the framework, multi-center prospective studies are needed. Independent external validation using data from different healthcare institutions and geographical regions is important for assessing whether benchmark results also hold in clinical practice. The validation should include diverse patient populations. Reporting should also cover discrimination, calibration, and clinically relevant outcomes in a consistent manner.

#### 4.2.5 Explainability and clinical trust

Explainability remains important for clinical use because clinicians need to know whether model predictions are based on meaningful clinical information. Although the reviewed studies often used post-hoc explanations, such as Grad-CAM, attention-based explanations, saliency maps, and feature attribution, quantitative assessment of explanation quality was less common (28, 64). As a result, it is challenging to determine whether explanations that initially appear reasonable truly represent the variables influencing the model’s predictions. Standardized evaluation frameworks that assess both localization accuracy and clinical relevance are therefore needed (82). Explainability alone may not be sufficient for clinical use. Uncertainty estimation and model calibration are also important. A model can produce a high-confidence result when it is tested on an unfamiliar patient population or a different imaging distribution. Uncertainty estimation can help identify cases where the result may be less reliable. Calibration indicates whether the predicted probabilities correspond to the actual likelihood of an outcome. Future multimodal pneumonia studies should therefore report calibration measures and, when appropriate, uncertainty estimates along with standard measures such as accuracy and AUC.

### 4.3 Emerging artificial intelligence trends

The studies included in this review show that traditional task-specific modelling approaches are gradually giving way to scalable, efficient, and multimodal AI applications. The emerging approaches discussed in this section may support multimodal pneumonia diagnosis by addressing some of the limitations of current methods. Foundation and pretrained models, self-supervised learning, vision-language learning, federated learning, and generative artificial intelligence are some of the study areas that are now gaining greater attention. They all offer ways to get around many of the current restrictions associated with the concepts and techniques used today. In particular, they offer the opportunity to reduce reliance on large volumes of annotated data, leverage fragmented multimodal data, address inter-institutional variation in healthcare institutions, and improve generalizability. For deployment, these developments should be supported by reliable uncertainty estimates, proper calibration, and evaluation of explanation quality. Predictive performance alone is not sufficient.

#### 4.3.1 Foundation and pretrained models

Large pretrained models have shown considerable potential for improving pneumonia diagnosis by transferring knowledge learned from large-scale datasets to smaller clinical datasets. Pretrained feature embeddings may be useful in chest X-ray evaluation, particularly in situations where labelled pneumonia data is scarce, according to some recent research employing transformer-based and transfer-learning techniques (24, 83, 84). To reduce the requirement for task-specific data annotation, foundation models trained on a variety of data sources, including medical images, clinical notes, lab findings, and clinical language, may be used in the future. These models will work well in a variety of healthcare environments and pneumonia subtypes. Overall, the reviewed literature highlights large-scale multimodal pretraining as a promising direction for reducing dependence on labelled datasets while improving the flexibility and generalizability of future AI systems.

#### 4.3.2 Vision-language models

Using medical images and related clinical/radiological data, vision-language models offer a promising new approach to improving pneumonia detection. The usefulness of using language in vision-based pneumonia detection is demonstrated by the success of using language-driven attention in recent transformer systems (24). The development of multimodal representation learning, report generation, and poorly supervised illness diagnosis has been made possible by the availability of large paired image-report databases, such as MIMIC-CXR (70). However, before using such models in the healthcare industry, several problems must be resolved. These include inadequate supervision, the possibility of fraudulent results, ambiguity in clinical reports, and discrepancies between radiological findings and reports.

#### 4.3.3 Self-supervised learning

Medical image analysis is increasingly focusing on self-supervised learning since it allows learning on unlabelled data before fine-tuning on smaller labelled datasets. To support weakly supervised and multimodal techniques, some of the reviewed studies have used existing open-access databases such as CheXpert and MIMIC-CXR, where chest X-ray images are annotated using clinical reports (69, 70). Jobayer et al. (85) investigated a progressive semi-supervised approach for pneumonia diagnosis using respiratory sound data. Their method used three types of representations: time-series, Mel-spectrogram, and wavelet features. The results suggest that this combination of semi-supervised and multi-representation learning may reduce the need for large amounts of labeled clinical data. Only a few numbers of them have been evaluated in various clinical settings, despite their encouraging results. Further investigation is required to determine the effectiveness of self-supervised pre-training in conjunction with transformer-based and multimodal methods for pneumonia detection.

#### 4.3.4 Federated learning

Pneumonia diagnostic models could be developed using the federated learning approach without requiring the transfer of patient data between healthcare facilities. However, this approach still has certain issues, according to the literature assessment. The model’s accuracy and training process may be affected by patient heterogeneity, non-identically distributed (non-IID) data distribution, annotation techniques, and network connectivity. Furthermore, according to some experts, safeguarding data privacy required actions beyond federated learning. Therefore, further research is needed to confirm federated learning before putting it into practice, in spite its potential (86).

#### 4.3.5 Generative artificial intelligence

Generative models have the potential to support pneumonia diagnosis by creating synthetic medical images, improving representation of underrepresented classes, and reducing problems related to class imbalance. For example, the Vi-GeN 1.0 framework, which combined the Vision Transformer with a Wasserstein generative adversarial network to create chest X-rays, exhibited strong classification performance and increased dataset variety (87). Furthermore, variational autoencoders and generative adversarial networks have been investigated for their potential use in handling limited samples of minority pneumonia classes (88). However, using fabricated data requires careful consideration. Instead of focusing on illness patterns, artificial samples should be assessed based on their clinical plausibility, diversity, privacy concerns, and potential replication of dataset-specific abnormalities.

In conclusion, the aforementioned cutting-edge techniques may contribute to the development of multimodal AI solutions for pneumonia that go beyond the existing limited and privacy-insecure unimodal classifiers. It is necessary to verify the dependability of these techniques on a variety of patient samples before implementing them in routine clinical practice.

### 4.4 Future research directions and technology roadmap

It appears that merely increasing AI systems’ diagnostic accuracy would not be sufficient for their application in clinical settings, based on the results of this research review. Future study should also address issues with data heterogeneity, external validation, interpretability, and workflow integration, all of which have been identified as recurrent issues in the publications this review looked at.

#### 4.4.1 Development of large-scale multimodal datasets

The development of large multimodal databases is one of the main objectives for upcoming pneumonia AI research. Most current literature reviews rely on single-centre databases, which cannot be diverse or have all the required data modalities (15–17). The creation of comprehensive benchmark databases that incorporate medical imaging, laboratory testing, clinical notes, respiratory sound recordings, and molecular data is necessary. They would make it easier to apply different models in clinical practice and enable a thorough evaluation of their performance.

#### 4.4.2 Robust multimodal learning with missing modalities

Because patients do not complete all diagnostic tests, many datasets are incomplete. Future multimodal AI systems must therefore be created to guarantee their efficacy even in the absence of certain data types, such as imaging, lab, clinical text, and genomic data (48, 49). Developing techniques for robust learning, adaptive fusion, and uncertainty awareness will be crucial to improving the usefulness of AI systems.

#### 4.4.3 Intrinsically explainable artificial intelligence

Future research should focus on developing intrinsically interpretable multimodal models that would explicitly describe interactions between imaging data, clinical data, laboratory test results, and respiratory data, despite advancements in post-hoc explainability tools like Grad-CAM, SHAP, and attention visualization techniques that make deep learning models more transparent (28, 64). Frameworks for evaluating the quality and clinical relevance of produced explanations are also required. Future studies should also examine uncertainty and calibration to determine whether the model’s confidence is reliable, particularly when it encounters cases that differ from the training data. This will support safe use of AI-based diagnostic systems, build physicians’ confidence in the results, and inform clinical and regulatory evaluation.

#### 4.4.4 Prospective multi-center clinical validation

The majority of published research has used retrospective, single-centre datasets, which limits the findings’ applicability in real-world clinical settings (8, 25, 26). Future research must focus on validating the model in a prospective multi-center environment with a variety of patients, evaluation techniques, and performance metrics. This will reduce domain differences, allow for assessing the models’ effectiveness, and make it easier for AI to be included into clinical decision-making.

#### 4.4.5 Personalized and adaptive pneumonia diagnosis

By combining imaging results, clinical data, laboratory data, respiratory data, and genomic data, future AI systems would allow for personalised diagnosis of pneumonia. The process of diagnosis, disease categorization, prognosis prediction, and therapy recommendations can be improved by new approaches such as foundation models, vision-language models, self-supervised learning, federated learning, and generative AI (83). The aspects that must be taken into account for future multimodal AI pneumonia diagnosis include data collection techniques, effective integration of many modalities, model interpretability, clinical verification, and individualised decision support. The aforementioned fields can aid in developing efficient AI systems for pneumonia diagnosis.

### 4.5 Limitations

This systematic review focuses on recent developments in artificial intelligence, particularly multimodal deep learning and fusion algorithms, for pneumonia diagnosis. The PRISMA 2020 guidelines were followed to keep the study selection process transparent. However, several limitations should be acknowledged. First, the review was limited to English-language, peer-reviewed journal articles. Relevant studies published in other languages or publication formats may therefore have been missed. Second, although Scopus, PubMed, IEEE Xplore, and Web of Science were searched, some relevant studies may not have been identified because of differences in database coverage. The included studies also varied in terms of data modalities, model architectures, fusion techniques, datasets, and model evaluation methods. These differences made a quantitative meta-analysis of the findings was not feasible. Since there was no way to compare the data meaningfully using statistics, the results were synthesized qualitatively. Variations in validation strategies and reporting practices may also affect the assessment of model generalizability and clinical applicability. Finally, publication bias cannot be completely excluded. Another limitation concerns differences in datasets and validation procedures across the studies. Many models were tested using retrospective, single-center, or benchmark datasets that may not represent patients seen in routine clinical practice. Patient populations, imaging protocols, and annotation practices also varied between studies. Such differences can lead to dataset bias and domain shift, which may affect how well the models perform in other clinical settings. For this reason, strong results on benchmark datasets should not be considered evidence of clinical utility, particularly when independent external or prospective validation is lacking.

### 4.6 Conclusion

This systematic review provides an overview of recent advances in deep learning for pneumonia diagnosis. Multi-modal AI architectures that include various healthcare information sources are given particular attention. Overall, the reviewed studies suggest that multimodal approaches can benefit from information obtained from different healthcare data sources. In some cases, this may lead to better diagnostic performance and model robustness. Direct comparison with single-modality approaches is difficult, however, because the studies vary in their datasets, patient populations, clinical tasks, model architectures, evaluation metrics, and validation methods. For this reason, higher performance reported by some multimodal models should not be considered proof that they are consistently better than single-modality models in all clinical settings. The examined articles also demonstrate explainable AI techniques improve model transparency and support clinical interpretation. Nevertheless, several issues still need to be resolved before these technologies may be used in medical practice. These include the absence of large-scale multi-modal datasets, data heterogeneity, missing modalities, limited interpretability, and a lack of external validation. Overall, this review presents a broad framework for examining healthcare data modalities, explainability techniques, multimodal fusion approaches, deep learning architectures, and benchmark datasets. Additionally, it draws attention to the gaps in the current research and the areas that still need to be investigated. Using these findings, multimodal AI algorithms for the identification of pneumonia can be developed that are robust and clinically useful.

## References

[1] World Health Organization. Data from: Pneumonia (2023).

[2] Arnold SR Jain S Dansie D Kan H Williams DJ Ampofo K, et al. Association of radiology findings with etiology of community acquired pneumonia among children. J Pediatr. (2023) 261:113333. 10.1016/j.jpeds.2023.01.010 36736585 PMC12798854

[3] Senthil Kumar J Pradeepa R Arulkarthick D Chandragandhi S. Prediction of the pneumonia from the CT lung images by using the multiband google NET CNN. Biomed Signal Process Control. (2025) 107:107738. 10.1016/j.bspc.2025.107738

[4] Wahyuningrum R Bauravindah A Siradjuddin I Satoto B Kartikasari A Sensusiati A. Enhancing lung disease diagnosis with deep-learning-based CT scan image segmentation. Intell Syst Appl. (2025) 27:200565. 10.1016/j.iswa.2025.200565

[5] Moure-Prado A Guerrero-López A Arias-Londoño J Godino-Llorente J. Bayesian automatic screening of pneumonia and lung lesions localization from CT scans: a combined method toward a more user-centred and explainable approach. IEEE Access. (2025) 13:178160–75. 10.1109/ACCESS.2025.3607282

[6] Ju F Zhao S Zhu S. Enhanced pneumonia lesion segmentation using a hybrid CNN-BiFormer network with residual haar wavelet downsampling and shared attention. Multimed Syst. (2025) 31:409. 10.1007/s00530-025-01990-4

[7] Yu Y Bai H Chen M Yang L Zhang B Wang J, et al. WSDC-ViT: a novel transformer network for pneumonia image classification based on windows scalable attention and dynamic rectified linear unit convolutional modules. Sci Rep. (2025) 15:27868. 10.1038/s41598-025-12117-0 40739309 PMC12310959

[8] Guo K Cheng J Li K Wang L Lv Y Cao D. Diagnosis and detection of pneumonia using weak-label based on X-ray images: a multi-center study. BMC Med Imaging. (2023) 23:209. 10.1186/s12880-023-01174-4 38087255 PMC10717871

[9] Wang X Peng Y Lu L Lu Z Bagheri M Summers RM. ChestX-Ray8: hospital-scale chest X-ray database and benchmarks on weakly-supervised classification and localization of common thorax diseases. In: *2017 IEEE conference on computer vision and pattern recognition (CVPR)* (Honolulu, HI: IEEE) (2017), 3462–71. 10.1109/CVPR.2017.369

[10] Rajpurkar P Irvin J Zhu K Yang B Mehta H Duan T, et al. CheXNet: radiologist-level pneumonia detection on chest X-rays with deep learning (2017). 10.48550/ARXIV.1711.05225

[11] Kundu R Das R Geem ZW Han GT Sarkar R. Pneumonia detection in chest X-ray images using an ensemble of deep learning models. PLoS One. (2021) 16:e0256630. 10.1371/journal.pone.0256630 34492046 PMC8423280

[12] Shakeri E Far B. Advancing pneumonia classification and detection: comparative analysis of deep learning models using convolutional neural networks. In: *2024 IEEE international conference on information reuse and integration for data science (IRI)* (San Jose, CA, United States: IEEE) (2024), 108–13. 10.1109/IRI62200.2024.00033

[13] Moradi M Hassanzadeh A Haghanifar A Ko S. Optimized COVID-19 detection using sparse deep learning models from multimodal imaging data. Multimed Tools Appl. (2025) 84:11451–74. 10.1007/s11042-024-18987-2

[14] Li J Luo X Ma H Zhao W. A hybrid deep transfer learning model with Kernel metric for COVID-19 pneumonia classification using chest CT images. IEEE/ACM Trans Comput Biol Bioinform. (2023) 20:2506–17. 10.1109/TCBB.2022.3216661 36279353

[15] Hong Y Chen L Yu Y Zhao Z Wu R Gong R, et al. Deep learning integration of chest computed tomography and plasma proteomics to identify novel aspects of severe COVID-19 pneumonia. J Intensive Med. (2025) 5:252–61. 10.1016/j.jointm.2024.11.001 40933743 PMC12417366

[16] Wang Y Liu C Fan Y Niu C Huang W Pan Y, et al. A multi-modal deep learning solution for precise pneumonia diagnosis: the PneumoFusion-Net model. Front Physiol. (2025) 16:1512835. 10.3389/fphys.2025.1512835 40144549 PMC11937601

[17] Xing W Li M Liu Y Liu X Li Y Yang Y, et al. Multi-omics graph knowledge representation for pneumonia prognostic prediction. IEEE J Biomed Health Inform. (2025) 29:3021–34. 10.1109/JBHI.2024.3488735 39475733

[18] Bikku T Kpnv S Thota S Pujari J Batchu R Mortezaagha P, et al. Deep learning-driven early diagnosis of respiratory diseases using CNN-RNN fusion on lung sound data. Sci Rep. (2025) 15:45233. 10.1038/s41598-025-28832-7 41286281 PMC12748899

[19] Lucassen RT Jafari MH Duggan NM Jowkar N Mehrtash A Fischetti C, et al. Deep learning for detection and localization of B-lines in lung ultrasound. IEEE J Biomed Health Inform. (2023) 27:4352–61. 10.1109/JBHI.2023.3282596 37276107 PMC10540221

[20] Page MJ McKenzie JE Bossuyt PM Boutron I Hoffmann TC Mulrow CD, et al. The PRISMA 2020 statement: an updated guideline for reporting systematic reviews. BMJ. (2021) 372:n71. 10.1136/bmj.n71 33782057 PMC8005924

[21] Li D. Attention-enhanced architecture for improved pneumonia detection in chest X-ray images. BMC Med Imaging. (2024) 24:6. 10.1186/s12880-023-01177-1 38166579 PMC10763425

[22] Wang T Nie Z Wang R Xu Q Huang H Xu H, et al. PneuNet: deep learning for COVID-19 pneumonia diagnosis on chest X-ray image analysis using vision transformer. Med Biol Eng Comput. (2023a) 61:1395–408. 10.1007/s11517-022-02746-2 PMC9887581 36719562

[23] Sun J Xue J Xu Z Li N Tang C Zhao L, et al. SAGCN: self-adaptive graph convolutional network for pneumonia detection. Biomed Signal Process Control. (2025) 106:107634. 10.1016/j.bspc.2025.107634

[24] Chen Q Chen L Nie W Li X Zheng J Zhong J, et al. A mixed-scale dynamic attention transformer for pediatric pneumonia diagnosis. Displays. (2025) 87:102953. 10.1016/j.displa.2024.102953

[25] Okolo G Katsigiannis S Ramzan N. CLN: a multi-task deep neural network for chest X-ray image localisation and classification. Expert Syst Appl. (2025) 288:128162. 10.1016/j.eswa.2025.128162

[26] Akshay A Kashyap S Patel HND Kumar RP. Hybrid deep learning and machine learning framework for automated pneumonia detection in chest X-ray images. MethodsX. (2025) 15:103729. 10.1016/j.mex.2025.103729 41404541 PMC12704267

[27] Fu M Tantithamthavorn C Le T. DAViT: a domain-adapted vision transformer for automated pneumonia detection and explanation using chest X-ray images. IEEE Access. (2025) 13:103033–44. 10.1109/ACCESS.2025.3579314

[28] Fu Y Xue P Zhang Z Dong E. PKA2-Net: prior knowledge-based active attention network for accurate pneumonia diagnosis on chest X-ray images. IEEE J Biomed Health Inform. (2023) 27:3513–24. 10.1109/JBHI.2023.3267057 37058372

[29] Liu W Ni Z Chen Q Ni L. Attention-guided partial domain adaptation for automated pneumonia diagnosis from chest X-ray images. IEEE J Biomed Health Inform. (2023) 27:5848–59. 10.1109/JBHI.2023.3313886 37695960

[30] Wang F Li X Wen R Luo H Liu D Qi S, et al. Pneumonia-plus: a deep learning model for the classification of bacterial, fungal, and viral pneumonia based on CT tomography. Eur Radiol. (2023) 33:8869–78. 10.1007/s00330-023-09833-4 37389609

[31] Scapicchio C Chincarini A Ballante E Berta L Bicci E Bortolotto C, et al. A multicenter evaluation of a deep learning software (LungQuant) for lung parenchyma characterization in COVID-19 pneumonia. Eur Radiol Exp. (2023) 7:18. 10.1186/s41747-023-00334-z 37032383 PMC10083148

[32] Demsash AW Abebe R Gezimu W Kitil GW Tizazu MA Lambebo A, et al. Data-driven machine learning algorithm model for pneumonia prediction and determinant factor stratification among children aged 6–23 months in Ethiopia. BMC Infect Dis. (2025) 25:647. 10.1186/s12879-025-10916-4 40316929 PMC12048943

[33] M’hamdi Z Lazaar M Mahboub O. Leveraging pretrained embeddings and self-attention based convolutional neural networks for early prediction of ventilator-associated pneumonia from clinical notes. Eng Appl Artif Intell. (2025) 154:110827. 10.1016/j.engappai.2025.110827

[34] Zhou J Ren X Gao J Wu R Chen L Li Z. Amplification-free detection of mycoplasma pneumoniae via CRISPR-Cas12a and deep learning-optimized crRNAs on a lateral flow platform. J Pharm Biomed Anal. (2026) 268:117196. 10.1016/j.jpba.2025.117196 41129856

[35] Shehab SA Mohammed KK Darwish A Hassanien AE. Deep learning and feature fusion-based lung sound recognition model to diagnoses the respiratory diseases. Soft comput. (2024) 28:11667–83. 10.1007/s00500-024-09866-x

[36] Huang D Wang L Wang W. A multi-center clinical trial for wireless stethoscope-based diagnosis and prognosis of children community-acquired pneumonia. IEEE Trans Biomed Eng. (2023) 70:2215–26. 10.1109/TBME.2023.3239372 37021995

[37] Sharan R Qian K Yamamoto Y. Automated cough sound analysis for detecting childhood pneumonia. IEEE J Biomed Health Inform. (2024) 28:193–203. 10.1109/JBHI.2023.3327292 37889830

[38] Jones BE Ramirez JA Oren E Soni NJ Sullivan LR Restrepo MI, et al. Diagnosis and management of community-acquired pneumonia. An official american thoracic society clinical practice guideline. Am J Respir Crit Care Med. (2025) 212:rccm.202507-1692ST. 10.1164/rccm.202507-1692ST 40679934

[39] Yen CT Tsao CY. Lightweight convolutional neural network for chest X-ray images classification. Sci Rep. (2024) 14:29759. 10.1038/s41598-024-80826-z 39613790 PMC11607463

[40] Yang Z Chen S Tang X Wang J Liu L Hu W, et al. Development and validation of machine learning-based prediction model for severe pneumonia: a multicenter cohort study. Heliyon. (2024) 10:e37367. 10.1016/j.heliyon.2024.e37367 39296114 PMC11408761

[41] Peng X Liu Y Zhang B Yang C Dong J Yong C, et al. A preliminary prediction model of pediatric mycoplasma pneumoniae pneumonia based on routine blood parameters by using machine learning method. BMC Infect Dis. (2024) 24:707. 10.1186/s12879-024-09613-5 39026207 PMC11264635

[42] Gupta R Singh R Travieso-González CM Burget R Kishore Dutta M. DeepRespNet: a deep neural network for classification of respiratory sounds. Biomed Signal Process Control. (2024) 93:106191. 10.1016/j.bspc.2024.106191

[43] Vyas R Khadatkar D. Ensemble of deep learning architectures with machine learning for pneumonia classification using chest X-rays. J Imaging Inform Med. (2025) 38:727–46. 10.1007/s10278-024-01201-y 39138748 PMC11950602

[44] Barman J Giri AK. Ensemble localized patch residual convolution neural networks for pneumonia detection using chest X-ray. BMC Med Inform Decis Mak. (2026) 26:213. 10.1186/s12911-026-03497-y 42046054 PMC13267658

[45] Abdullah Ather MA Sardar K Fatima Z Rodríguez JLO Sánchez-Mejorada CG, et al. HybridCATNet: an explainable CNN–transformer framework with early multi-channel fusion and uncertainty estimation for pneumonia detection from chest X-rays. Front Digit Health. (2026) 8:1769444. 10.3389/fdgth.2026.1769444 42564731 PMC13445601

[46] Jeon ET Lee HJ Park TY Jin KN Ryu B Lee HW, et al. Machine learning-based prediction of in-ICU mortality in pneumonia patients. Sci Rep. (2023) 13:11527. 10.1038/s41598-023-38765-8 37460837 PMC10352276

[47] Bukenya L Eyobu OS Oyana TJ. An optimized LSTM model for diagnosis prediction of lower respiratory tract infections using a minimalistic data set. IEEE Access. (2025) 13:60341–54. 10.1109/ACCESS.2025.3540359

[48] Wang Y Rao Y Zhu Y Wu J Qiao B Wu X, et al. Multimodal-based auxiliary diagnosis for pediatric community acquired pneumonia. Sci Rep. (2025) 15:39152. 10.1038/s41598-025-23006-x 41203666 PMC12594957

[49] Ren H Jing F Chen Z He S Zhou J Liu L, et al. CheXMed: a multimodal learning algorithm for pneumonia detection in the elderly. Inf Sci. (2024) 654:119854. 10.1016/j.ins.2023.119854

[50] VS P KK T. Enhancing COVID-19 detection through multimodal CT and X-ray image fusion with anisotropic diffusion and loss-attentional physics-informed neural networks. Biomed Signal Process Control. (2025) 110:108200. 10.1016/j.bspc.2025.108200

[51] Hofmeister J Garin N Platon A Scheffler M Montet X Poletti PA, et al. A multimodal AI model combining imaging, clinical and biological data significantly outperformed CXR-based AI diagnosis in pneumonia. Eur J Radiol Artif Intell. (2026) 6:100089. 10.1016/j.ejrai.2026.100089

[52] Fu Y Li Y Xu X Su C Xia H Song X. STAR-ViT: a spatially transformed and adversarially realigned vision transformer for pathogen classification in pediatric pneumonia. IEEE J Biomed Health Inform. (2026) 1–14. 10.1109/JBHI.2026.3713299 42447010

[53] Yang Y Mei G Piccialli F. A deep learning approach considering image background for pneumonia identification using explainable AI (XAI). IEEE/ACM Trans Comput Biol Bioinform. (2024) 21:857–68. 10.1109/TCBB.2022.3190265 35820002

[54] Lan XH Zhang YX Yuan WH Shi F Guo WL. Image-based deep learning in diagnosing mycoplasma pneumonia on pediatric chest X-rays. BMC Pediatr. (2024) 24:720. 10.1186/s12887-024-05204-0 39529076 PMC11552354

[55] Rabbah J Ridouani M Hassouni L. Improving pneumonia diagnosis with high-accuracy CNN-based chest X-ray image classification and integrated gradient. Biomed Signal Process Control. (2025) 101:107239. 10.1016/j.bspc.2024.107239

[56] Wang X Yu J Lin Y Yao B Qiu Z Zhan Y, et al. A multi-branch dual residual attention CNN model for pediatric pneumonia classification. Big Data Min Anal. (2026) 9:160–77. 10.26599/BDMA.2025.9020066

[57] Li Y Zhang L Yu H Wang J Wang S Liu J, et al. A comprehensive segmentation of chest X-ray improves deep learning–based WHO radiologically confirmed pneumonia diagnosis in children. Eur Radiol. (2024) 34:3471–82. 10.1007/s00330-023-10367-y 37930411

[58] Liu T Zhang ZH Zhou QH Cheng QZ Yang Y Li JS, et al. MI-DenseCFNet: deep learning–based multimodal diagnosis models for Aureus and Aspergillus pneumonia. Eur Radiol. (2024) 34:5066–76. 10.1007/s00330-023-10578-3 38231392 PMC11254966

[59] Huang D Gong L Wei C Wang X Liang Z. An explainable machine learning-based model to predict intensive care unit admission among patients with community-acquired pneumonia and connective tissue disease. Respir Res. (2024) 25:246. 10.1186/s12931-024-02874-3 38890628 PMC11186131

[60] Chen J Hou D Song Y. Development and multi-database validation of interpretable machine learning models for predicting In-Hospital mortality in pneumonia patients: A comprehensive analysis across four healthcare systems. Respir Res. (2025) 26:279. 10.1186/s12931-025-03348-w 41029388 PMC12486837

[61] Li J Zhang Y He S Tang Y. Interpretable mortality prediction model for ICU patients with pneumonia: using shapley additive explanation method. BMC Pulm Med. (2024) 24:447. 10.1186/s12890-024-03252-x 39272037 PMC11395639

[62] Shaikh M Ali Arain Q Jaffari R. AG-FSNet: a hybrid attention and feature selection network with feature contribution scoring for pneumonia segmentation using CXR images. Biomed Signal Process Control. (2026) 114:109248. 10.1016/j.bspc.2025.109248

[63] Ayub F Javid I Khattak MK Hayyat A. Beyond accuracy: a clinically safe, uncertainty-aware EfficientNetB3 framework for interpretable pneumonia screening. Biomed Signal Process Control. (2026) 126:110852. 10.1016/j.bspc.2026.110852

[64] Slimi H Balti A Abid S Sayadi M. Trustworthy pneumonia detection in chest X-ray imaging through attention-guided deep learning. Sci Rep. (2025) 15:40029. 10.1038/s41598-025-23664-x 41238604 PMC12618509

[65] Hofmeister J Garin N Montet X Scheffler M Platon A Poletti PA, et al. Validating the accuracy of deep learning for the diagnosis of pneumonia on chest x-ray against a robust multimodal reference diagnosis: a post hoc analysis of two prospective studies. Eur Radiol Exp. (2024) 8:20. 10.1186/s41747-023-00416-y 38302850 PMC10834924

[66] Shih G Wu CC Halabi SS Kohli MD Prevedello LM Cook TS, et al. Augmenting the national institutes of health chest radiograph dataset with expert annotations of possible pneumonia. Radiol Artif Intell. (2019) 1:e180041. 10.1148/ryai.2019180041 33937785 PMC8017407

[67] Wu L Zhang J Wang Y Ding R Cao Y Liu G, et al. Pneumonia detection based on RSNA dataset and anchor-free deep learning detector. Sci Rep. (2024) 14:1929. 10.1038/s41598-024-52156-7 38253758 PMC10803753

[68] Kermany DS Goldbaum M Cai W Valentim CC Liang H Baxter SL, et al. Identifying medical diagnoses and treatable diseases by image-based deep learning. Cell. (2018) 172:1122–31.e9. 10.1016/j.cell.2018.02.010 29474911

[69] Irvin J Rajpurkar P Ko M Yu Y Ciurea-Ilcus S Chute C, et al. Data from: CheXpert: a large chest radiograph dataset with uncertainty labels and expert comparison (2019). 10.48550/ARXIV.1901.07031

[70] Johnson AEW Pollard TJ Berkowitz SJ Greenbaum NR Lungren MP Deng C, et al. MIMIC-CXR, a de-identified publicly available database of chest radiographs with free-text reports. Sci Data. (2019) 6:317. 10.1038/s41597-019-0322-0 31831740 PMC6908718

[71] Serbes G Ulukaya S Kahya YP, An automated lung sound preprocessing and classification system based on spectral analysis methods. In: *International Conference on Biomedical and Health Informatics 2017 Nov 17*, Singapore: Springer Singapore (2017). pp. 45–9. 10.1007/978-981-10-7419-6_8

[72] Wang Z Wang Y Sun Z. Sensitivity analysis of data augmentation methods on performance of deep learning model for lung sounds classification. Sci Rep. (2025) 15:39268. 10.1038/s41598-025-23106-8 41214170 PMC12603304

[73] Sharma N Krishnan P Kumar R Ramoji S Chetupalli SR Nirmala R, et al. Coswara—a database of breathing, cough, and voice sounds for COVID-19 diagnosis. In: *Interspeech 2020* (ISCA) (2020), 4811–5. 10.21437/Interspeech.2020-2768

[74] Johnson AEW Bulgarelli L Shen L Gayles A Shammout A Horng S, et al. MIMIC-IV, a freely accessible electronic health record dataset. Sci Data. (2023) 10:1. 10.1038/s41597-022-01899-x 36596836 PMC9810617

[75] Johnson A. Data from: alistairewj/mimic-iv-aline-study: MIMIC-IV aline study v0.1 (2021). 10.5281/ZENODO.5579577

[76] Xie S Wu M Shang Y Tuo W Wang J Cai Q, et al. Development and validation of an early diagnosis model for severe mycoplasma pneumonia in children based on interpretable machine learning. Respir Res. (2025) 26:182. 10.1186/s12931-025-03262-1 40361124 PMC12070602

[77] Anbukkarasi S Hemalatha S Balakrishnan A Varadhaganapathy S Easwaramoorthy SV. K-STAMM: a knowledge-enhanced spatial—temporal attention model with multimodal fusion for pneumonia prediction. Sci Rep. (2026) 16:16773. 10.1038/s41598-026-47146-w 41957440 PMC13223296

[78] Jin Y Zheng J Qu J Ren C Wang T Gan Y, et al. A deep learning-based multimodal data fusion algorithm for predicting checkpoint inhibitors related pneumonia. Biomed Signal Process Control. (2026) 118:109688. 10.1016/j.bspc.2026.109688

[79] Feng Y Huang G Ju F Cui H. Multimodal text guided network for chest CT pneumonia classification. Sci Rep. (2025) 15:32900. 10.1038/s41598-025-14165-y 40998864 PMC12464172

[80] Musa A Prasad R Hernandez M. Addressing cross-population domain shift in chest X-ray classification through supervised adversarial domain adaptation. Sci Rep. (2025) 15:11383. 10.1038/s41598-025-95390-3 40181036 PMC11968948

[81] Chandrashekar M Goethert I Haque MIU McMahon B Dhaubhadel S Knight K, et al. Domain shift analysis in chest radiographs classification in a veterans healthcare administration population. J Imaging Inform Med. (2026) 39:484–99. 10.1007/s10278-025-01494-7 40216674 PMC12921069

[82] Oltu B Güney S Yuksel S Dengiz B. Automated classification of chest X-rays: a deep learning approach with attention mechanisms. BMC Med Imaging. (2025) 25:71. 10.1186/s12880-025-01604-5 40038588 PMC11877751

[83] Kumar S Kumar H. PneuSwin: an advanced X-ray-based diagnostic system integrating ensemble deep learning architectures and swin transformers for pneumonia detection. J Sci Ind Res. (2025) 84:421–34. 10.56042/jsir.v84i04.14040

[84] Tripathy B Khan S Bebortta S Kamal A Tripathy S Fazil M, et al. TL-PneuNet: a transfer learning-based pneumonia classification framework. Sci Rep. (2025) 15:40307. 10.1038/s41598-025-24180-8 41249420 PMC12624121

[85] Jobayer M Shawon MMH Rasheduzzaman M Rahman MW Doulah A. Progressive semi-supervised learning for multimodal pneumonia diagnosis. Biomed Signal Process Control. (2026) 118:109750. 10.1016/j.bspc.2026.109750

[86] Pan Z Wang H Wan J Zhang L Huang J Shen Y. Efficient federated learning for pediatric pneumonia on chest X-ray classification. Sci Rep. (2024) 14:23272. 10.1038/s41598-024-74491-5 39375440 PMC11458595

[87] Dash A Panigrahi S Nayak D Dash A Swarnkar T. Vi-GeN 1.0: GAN-augmented vision transformer pipeline for diversified pneumonia classification. J Transform Technol Sustain Dev. (2025) 9:14. 10.1007/s41314-025-00081-6

[88] Alshanketi F Alharbi A Kuruvilla M Mahzoon V Siddiqui S Rana N, et al. Pneumonia detection from chest X-ray images using deep learning and transfer learning for imbalanced datasets. J Imaging Inform Med. (2025) 38:2021–40. 10.1007/s10278-024-01334-0 39557737 PMC12344030

